# Tyrosine kinases sample unique activation ensembles

**DOI:** 10.64898/2026.06.26.734786

**Published:** 2026-07-01

**Authors:** Yanchen Zhu, Matteo T. Degiacomi, Antonia S. J. S. Mey, Sukrit Singh

**Affiliations:** 1 EaStCHEM School of Chemistry, University of Edinburgh, David Brewster Road, Joseph Black Building, Edinburgh EH9 3FJ, United Kingdom; 2 School of Informatics, University of Edinburgh, 10 Crichton St, Edinburgh EH8 9AB, United Kingdom; 3 Computational and Systems Biology Program, Memorial Sloan Kettering Cancer Center, New York, NY 10065, USA

## Abstract

Protein kinase activation is driven by conformational changes across multiple structural components, including the conserved Asp–Phe–Gly (DFG) motif, but whether these transitions follow a universal mechanism remains unclear. Here we combine over 8.3 milliseconds of distributed unbiased molecular dynamics simulations with Markov state models (MSMs) to compare the conformational landscapes of the ABL1, EGFR and MET kinase domains. To maximize unbiased sampling of functionally relevant conformational space, we use a transfer seeding strategy that steers AlphaFold2 models derived from homologous templates to sample MET conformational states absent from available experimental databases. We find that related DFG-motif geometries separate into distinct kinetic networks. These shared structural states are connected by kinase-specific activation pathways with different regulatory elements controlling the slowest step of activation. Our findings reveal that the shared nomenclature masks distinct transition mechanisms between kinase domains, revealing new regions critical for activity and targetable conformations for inhibitor design.

## 1 Introduction

Protein kinases are central regulators of eukaryotic cellular signalling. More than 500 protein kinases are encoded in the human genome, accounting for approximately 2% of human genes (1). Kinases control diverse processes including cell growth, proliferation, differentiation, and apoptosis through phosphorylation of protein substrates (2; 3). Given their central role in all aspects of cell life, kinase activity is tightly regulated through mechanisms such as autoinhibition, phosphorylation, and allosteric modulation (4; 5; 6). Disruption of their activity by mutation or dysregulated expression is often associated with many human diseases, such as a variety of cancers, emphasizing kinases as one of the most important target classes in drug discovery (7; 8; 9; 10).

Kinase activity is largely rooted in its catalytic kinase domain (KD), a highly conserved domain across the protein kinase family that mediates phosphorylation activity through conformational changes. KDs have a bilobal structure, consisting of a smaller N-terminal lobe and a larger C-terminal lobe, with the ATP-binding site located in the cleft between them (Supplementary Fig. S2). Several conserved structural motifs have been found to play central roles in kinase catalysis and regulation (11). Briefly, the glycine-rich loop (G-loop) in the N-lobe helps position and stabilize ATP, and the *β*3 strand contains a conserved lysine that forms a salt bridge with the *α*C helix to organize the active-site architecture. In the C-lobe, the activation loop (A-loop) is a flexible segment that controls substrate access (12). At the N-terminus of the A-loop lies the highly conserved Asp-Phe-Gly (DFG) motif, whose Asp residue coordinates a divalent magnesium ion that helps position the ATP phosphates for catalysis (11; 13; 14; 15). Together, conserved elements organize the catalytic machinery required for phosphate transfer, and their coordinated rearrangements provide a structural basis by which kinases switch between functionally distinct conformational states (4; 11; 15).

**Figure 1:**
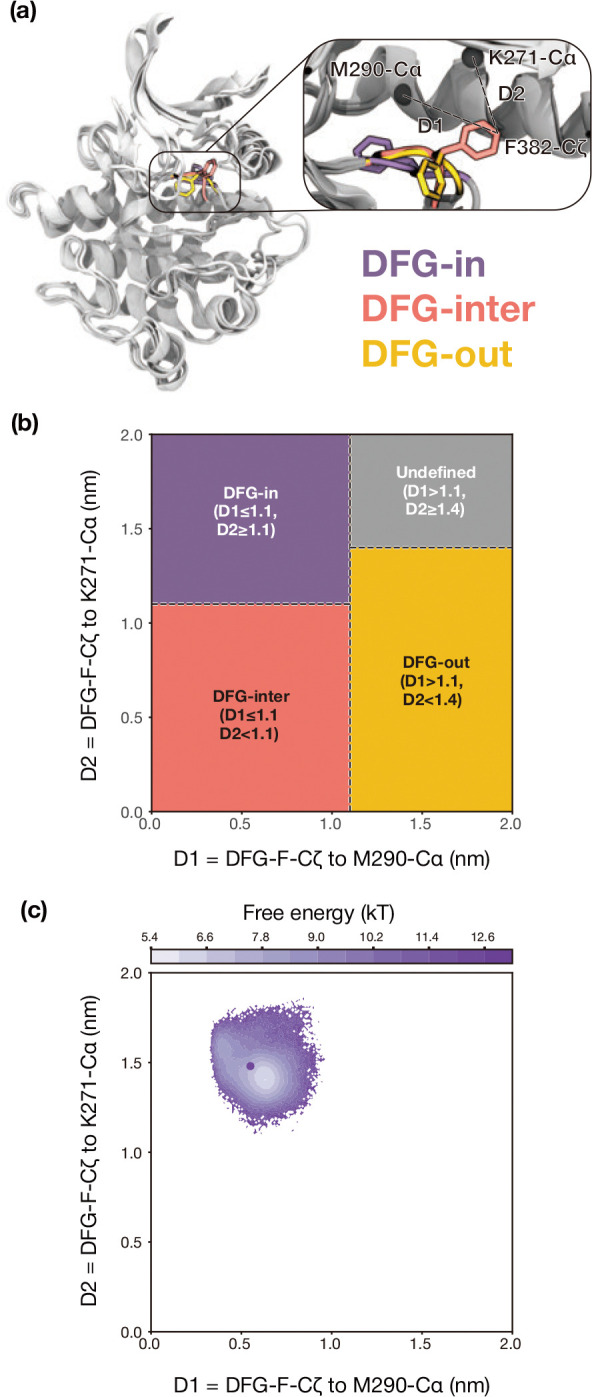
DFG motif conformational states of kinase domains. **(a)** Structural overlay of human ABL kinase in three conformations: DFG-in (PDB: 2f4j, purple), DFG-inter (PDB: 4xey, salmon), and DFG-out (PDB: 1opl, yellow). Inset: zoomed-in view of the DFG motif. D_1_ and D_2_ refer to distances between *α*C helix-M290-C*α* and DFG-F382-C*ζ*, and between *β*3-K271-C*α* and DFG-F382-C*ζ*, respectively. **(b)** Empirical partitioning of DFG states based on D_1_ and D_2_. **(c)** Aggregate 1235.39 *μ*s simulation initiated from a DFG-in conformation (PDB: 2wgj, purple point) projected onto D_1_ and D_2_, showing confinement to the DFG-in basin.

Kinase activity is not determined by a single static structure but by the dynamic equilibrium between active and inactive states. States are commonly distinguished by characteristic arrangements of the DFG motif, the *α*C helix, and the A-loop (Supplementary Fig. S2). Modi and Dunbrack have determined that three main conformational states—DFG-in, DFG-out, and DFG-inter—can be defined according to two distances between these structural elements (*D*_1_ and *D*_2_, Fig. 1b) (14; 16; 17). They further noted how these main states can be subdivided according to specific values of *φ*, *ψ* and *χ*_1_ angles within the DFG motif. The DFG-in BLAminus state corresponds most closely to the active kinase conformation where catalytic metal ions bind (see Methods) (14). Overall, the DFG motif is a central indicator for describing kinase conformational ensembles in which active-like, intermediate, and inactive-like states coexist.

Ultimately, kinase activity is determined by the relative populations of these aforementioned conformational states. (4; 17; 18; 19; 20). Changes in the active–inactive equilibrium are central to normal regulation, for example, through phosphorylation or ligand binding, but can also contribute to diseases. Indeed, several clinically observed kinase mutations bias the conformational ensemble towards the active state, resulting in dysregulated signalling and oncogenic activation (21; 22; 23). Conversely, kinase inhibitors exploit these conformational preferences through distinct binding modes (10; 24). Type I inhibitors bind competitively in the ATP pocket, whereas type II inhibitors extend into an adjacent hydrophobic back pocket that is typically exposed in the DFG-out inactive conformation. By stabilizing inactive states, type II inhibitors are broadly observed to achieve improved selectivity (25; 26). Allosteric inhibitors bind outside the ATP pocket and can stabilize distinct inactive or autoinhibited conformations, offering opportunities for high specificity and reduced resistance (27; 28). Thus, understanding how genetic variants, post-translational modifications, and ligand binding modulate kinase function requires a quantification of how these events and mutations modulate kinase structural ensembles (22; 29; 30; 31). Previous efforts have highlighted that some protein families follow common activation mechanisms, although it remains unclear if tyrosine kinase domains share a common activation ensemble (32). An ensemble-level characterization expands opportunities for structure-based drug design. The discovery of cryptic or transient states opens new opportunities for selective targeting to modulate kinase activity and potentially overcome resistance (33; 34; 35; 36; 37).

Despite their importance, kinase conformational ensembles remain challenging to characterize. Experimental techniques such as X-ray crystallography and cryo-EM provide high-resolution structural snapshots but typically capture static or averaged structures and commonly observe the most populated conformational states (38). Solution methods such as NMR and hydrogen–deuterium exchange can report on conformational dynamics but often provide limited atomistic detail for low-population intermediates (39). Molecular dynamics (MD) simulations provide a complementary route to probe kinase dynamics at atomic resolution, yet functionally important transitions, such as DFG flips and coupled A-loop or *α*C helix rearrangements, can occur on millisecond timescales and may remain inaccessible to conventional equilibrium simulations initiated from a single starting structure (15; 40; 41; 42; 43). Consistently, our own single-starting-point unbiased simulations struggle to sample the ensemble (Fig. 1c), showing that even 1.24 ms of unbiased simulations initiated from an experimentally determined DFG-in conformation remains trapped in this state. Enhanced-sampling methods can accelerate barrier crossing and discover rare states, but quantitative interpretation of the resulting ensembles can depend on the chosen collective variables, biasing protocol, and reweighting procedure (35; 44; 45; 46; 47). As a result, no singular protocol has observed multiple activation transitions across many kinase domains, and it remains unclear whether the activation pathway follows a conserved mechanism across kinases.

Markov state models (MSMs) provide a framework to address this challenge. By integrating information from multiple unbiased MD simulations, MSM analysis enables the identification of stable states and estimating transition timescales between them. Kinases are well-suited to this approach since many experimental structures spanning active and inactive states are available (12), providing seeds for sampling relevant conformational states (35; 48; 49; 50). However, for structurally under-represented kinases, experimental structures may not cover all relevant functional states. AI-based structure predictors offer a collective-variable-independent way to generate diverse structural seeds without first carrying out expensive exploratory simulations. In particular, AlphaFold2 (AF2) can be guided by template structures, enabling structural information from well-characterized homologues to be transferred onto a target sequence (51; 52; 53). For conserved protein families such as kinases and GPCRs, homologous structures annotated by conformational state can be used to generate physically plausible starting models in states that are poorly represented in the target’s experimental structural ensemble (43; 53; 54; 55). We refer to this strategy as transfer seeding, where homologue-derived conformational templates generate key target-specific seeds for subsequent unbiased MD and MSM analysis.

Here, we characterized the equilibrium conformational landscapes and activation pathways of three apo tyrosine kinase domains, ABL1 (which we refer to as ABL), EGFR, and MET, using MSMs constructed from unbiased MD simulations. We generated large aggregate unbiased simulation datasets comprising 0.592 ms for ABL, 1.836 ms for EGFR using PDB seeds, and 5.938 ms for MET using PDB and AF2-seeded structures on the Folding@home distributed computing platform (37) (Supplementary Fig. S6). By sampling each kinase with a consistent, unbiased simulation and MSM analysis protocol, our simulations enable direct comparison of their conformational ensembles. Although the three kinases sample similar active and inactive conformational states, we find that their equilibrium populations differ substantially. The three kinase activation pathways also proceed through distinct intermediate states and interconvert on timescales ranging from approximately 100 *μ*s to 6 ms. We further find that the A-loops define kinase-specific conformational ensembles that shape the transition pathways. Our work establishes a systematic biophysical framework for comparing kinase activation landscapes, enabling mechanistic discovery of how conserved structural elements give rise to kinase-specific conformational dynamics.

## 2 Results

### 2.1 ABL and EGFR share common DFG conformational states but differ in populations and kinetics

**Figure 2:**
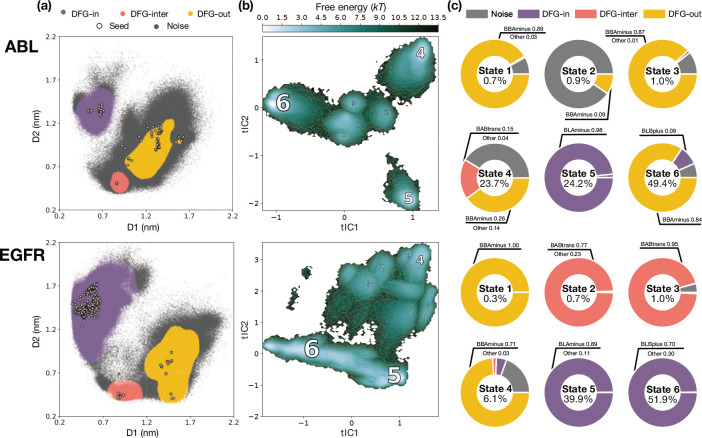
MSMs of ABL and EGFR explore similar DFG states but differ in population and landscapes. **(a)** DFG-state classification of MD simulation frames based on *D*_1_ and *D*_2_ distances. Each MD frame is a points coloured by assignment to DFG-in (purple), DFG-inter (salmon), DFG-out (yellow), or undefined (gray). Initial seeding structures are marked as white circles. **(b)** ABL and EGFR free energy landscapes estimated from MSM equilibrium distribution projected onto tIC1 and tIC2. Macrostates are labelled with sizes proportional to their populations. **(c)** Pie charts show the decomposition of each macrostate into Dunbrack states. Labels report macrostate equilibrium population and Dunbrack state fractions.

We extensively sampled kinase conformational ensembles using a diverse set of PDB seeds from two well-represented kinase domains, ABL and EGFR (Supplementary Table S1, Fig. S4, S5). Projection onto the *D*_1_ and *D*_2_ distances showed broad coverage of the DFG-in, DFG-inter, and DFG-out regions for both ABL and EGFR kinase domains (Fig. 2a, Fig. S4, S5), from which we built kinase-specific MSMs (Fig. 2b). We identified six macrostates in the ABL and EGFR MSMs, respectively (see Methods). To relate these macrostates to experimentally observed kinase conformations, we labelled them with the kinome-wide DFG motif classification introduced by Modi and Dunbrack (14) (see Methods), thereby comparing metastable states derived from MSMs with structural classes observed across the kinome.

Our MSMs show that ABL and EGFR access a shared set of conformational states, but assign different equilibrium populations to these shared states, which include the active-like DFG-in BLAminus state, the SRC-like inactive DFG-in BLBplus state (16; 56), the DFG-out BBAminus state, and the DFG-inter BABtrans state (Fig. 2c). In their respective six-state coarse-grained MSMs, most macrostates are dominated by a single Dunbrack class (Fig. 2c), indicating that the kinome-wide structural nomenclature captures a substantial part of the slow conformational heterogeneity resolved by the simulations. In apo ABL, the equilibrium ensemble is dominated by macrostate 6 (49.4%), which is primarily composed of DFG-out BBAminus conformations and resembles the inactive arrangement stabilized by type II inhibitors. The second most populated ABL macrostate, state 5 (24.2%), corresponds to the DFG-in BLAminus ensemble, which is characteristic of catalytically active kinase structures (17). The EGFR equilibrium ensemble is dominated by DFG-in conformations, with state 6 (51.9%) corresponding primarily to the BLBplus ensemble. This state adopts a SRC-like inactive conformation, in which the DFG-D remains oriented towards the ATP-binding site but the regulatory *β*3-Lys–*α*C-Glu salt bridge is disrupted and the A-loop is semi-closed compared with the active catalytic geometry (Supplementary Fig. S16). The second most populated EGFR macrostate 5 (39.9%) corresponds to the active-like BLAminus ensemble. Ensemble images of each macrostate are provided in Supplementary Figs. S8 and S9.

The correspondence between DFG structural labels and MSM macrostates is strong but not absolute. Specifically, we observe that kinetic proximity between macrostates cannot be directly inferred by DFG labels alone. In other words, the DFG labels are insufficient to predict what state any kinase domain is most likely to transition into, further highlighting the need for considering the complete ensemble. We quantified the interconversion timescales between macrostates using mean first-passage times (MFPTs), defined as the average time required for trajectories starting in one state to reach a target state for the first time (Fig. S13). In ABL, macrostate 6 contains both a DFG-out BBAminus substate and a DFG-in BLBplus substate. These two substates interconvert rapidly, with an MFPT of 68.2±0.20 *μ*s, whereas the transition from the BLBplus-containing macrostate 6 to the active-like BLAminus macrostate 5 occurs on a much slower timescale, with an MFPT of 2.39±0.20 ms (Supplementary Fig. S14). Therefore, the BBAminus-to-BLBplus transition represents a local, rapidly reversible rearrangement within this inactive basin. Similarly, ABL macrostate 4 contains kinetically mixed DFG-out BBAminus and DFG-inter BABtrans conformations (Supplementary Fig. S15). These conformations share an open A-loop but differ mainly through the dihedrals of DFG motifs and intermittently formed *β*3-Lys–*α*C-Glu salt bridge. We found this macrostate to resemble A-loop-closed structures bound to type I inhibitors in PDB (57; 58; 59). In summary, our MSM analysis shows that ABL and EGFR occupy a common DFG-centred conformational space, but differ substantially in how this space is thermodynamically populated and kinetically connected.

### 2.2 Transfer seeding expands coverage of the MET kinase DFG-state ensemble

**Figure 3:**
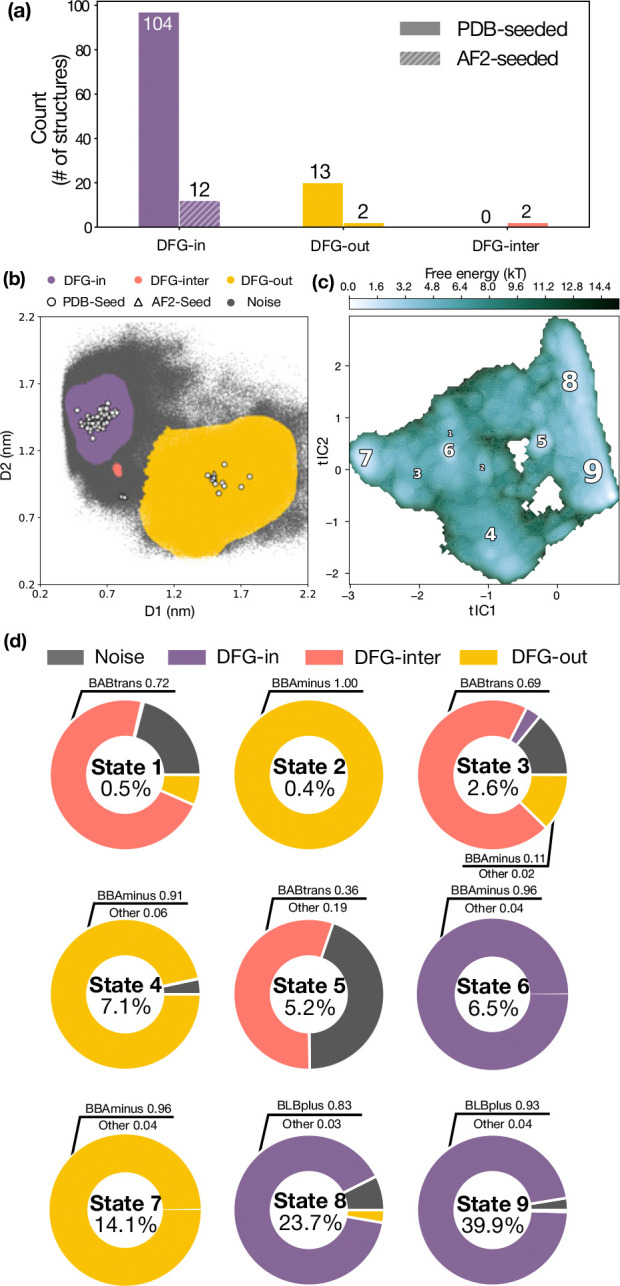
Complete sampling and MSM construction for MET kinase are enabled by transfer seeding. **(a)** Number of MET PDB structures (solid) and AF2-derived seeds (hatched) used for simulations. **(b)** Simulation snapshots projected onto *D*_1_ and *D*_2_ distances, coloured by DFG-state assignment as in Fig. 2. Initial seeding structures are marked to indicate PDB seeds (white circles) and AF-derived seeds (white triangles). **(c)** MET free energy landscape estimated from MSM equilibrium distribution projected onto tIC_1_ and tIC_2_. Macrostates are labelled with sizes proportional to their populations. **(d)** Pie charts show the decomposition of each macrostate into Dunbrack states. Labels report macrostate equilibrium population and Dunbrack state fractions.

MET is less represented in the experimental structural record (Fig. 3c, S17). Indeed, there are no MET DFG-inter structures available in the PDB, leaving a gap between the experimentally observed DFG-in and DFG-out regions (Fig. 3a, S17). Consistent with the rarity of DFG transitions on conventional MD timescales, simulations initiated only from this limited PDB ensemble are unlikely to identify missing intermediate states de novo.

We first tested whether missing MET DFG states could be recovered from the DFG-in basin using enhanced sampling approaches, including accelerated MD, Gaussian accelerated MD, umbrella sampling, metadynamics, and adaptive sampling (see Methods). Several of these approaches recovered DFG-inter and DFG-out conformations, indicating that the experimentally missing DFG-inter region is physically accessible. However, the apparent free-energy landscapes differed substantially between protocols, particularly in the relative depths of DFG basins and transition regions (Supplementary Fig. S1). We attribute this to a combination of biasing and reweighting uncertainty, incomplete barrier crossing, and limitations of low-dimensional collective variables. Meanwhile, some conformational changes sampled in unbiased MD involve coupled rearrangements and are not cleanly described as motion along *D*_1_ and *D*_2_ alone. Therefore, these biased simulations, without an in-depth consideration of multiple viable collective variables (54), are mainly for state-discovery, but are insufficient for quantitative estimates of the equilibrium ensemble. Details of enhanced sampling results can be found in the Supplementary Information.

To systematically generate a MET ensemble in a similar manner to ABL and EGFR without biasing physics, we built upon the principle of adaptive seeding where many parallel simulations are initialized from diverse conformations (35). Rather than relying on enhanced sampling to obtain seeds, we provided AF2 with annotated kinase structures from KinCore as conformational templates for MET modelling (see Methods, Supplementary Fig. S11). This template supervision was essential because untargeted AF2 modelling alone could not recover DFG states other than DFG-in (Supplementary Fig. S3). Notably, AF2 is most reliable at generating the desired templated conformation relative to other available co-folding methods (Supplementary Fig. S12).

We refer to this supervised structural generation as transfer seeding, in which structural information from ABL and EGFR is transferred onto the MET sequence with modern co-folding methods. Our transfer seeding approach successfully generates MET kinase structures in Dunbrack states not represented in the MET PDB ensemble and not obtainable by AF2 alone (Fig. 3a). To minimally augment the seeding of the MET kinase landscape without overly biasing the starting sampling away from experimental conformations, we provide only two structures per Dunbrack state (Fig. 3a).

Subsequent simulations and MSM analysis show that the absence of DFG-inter conformations in MET reflects incomplete experimental coverage rather than an inaccessible region of the MET conformational landscape. The union of PDB- and AF2-seeded simulations populated a connected DFG-in, DFG-inter, and DFG-out landscape (Fig. 3b). The resulting MET MSM shows that the DFG-inter conformations form part of this connected equilibrium conformational landscape with equilibrium population in states 1, 3, and 5 (Fig. 3c,d).

As with ABL and EGFR, Dunbrack state labels alone do not fully describe the MET conformational landscape because existing PDB structures may not capture the conformational heterogeneity of the A-loop. In the MET MSM, macrostates dominated by the same DFG label are not necessarily structurally equivalent (Fig. 3c,d). This is most apparent within the DFG-in ensemble, which separates into three distinct kinetic basins. State 6 (6.5%) corresponds to the active-like BLAminus ensemble, whereas states 9 (39.9%) and 8 (23.7%) both adopt BLBplus conformations but differ substantially in their A-loop ensembles (Supplementary Fig. S10). Similarly, DFG-out macrostates are all labelled as BBAminus but display distinct A-loop arrangements (Supplementary Fig. S10). Our results indicate that MET conformational heterogeneity extends beyond the DFG motif, with the A-loop contributing substantially to the separation of major kinetic basins within each DFG class.

MFPTs confirm that the structurally distinct macrostates 8 and 9 are kinetically separate from one another despite sharing the same Dunbrack state label (Supplementary Fig. S13). These states interconvert rapidly, with MFPTs of 12.0±0.6 *μ*s and 19.0±0.7 *μ*s in the forward and reverse directions, respectively, corresponding structurally to fast local A-loop exchange within the dominant DFG-in ensemble (Fig. S10). In contrast, transitions from states 8 and 9 to the active-like BLAminus state 6 occur on a much slower millisecond timescale, whereas escape from state 6 back to states 8 and 9 is approximately an order of magnitude faster. These observations indicate that the active-like MET basin is kinetically disfavoured in the apo form. However, we note that our understanding of these kinetic barriers is limited to the apo form and that the presence of cofactors and substrates may alter this transition probability.

### 2.3 Activation transitions follow kinase-specific pathways

**Figure 4:**
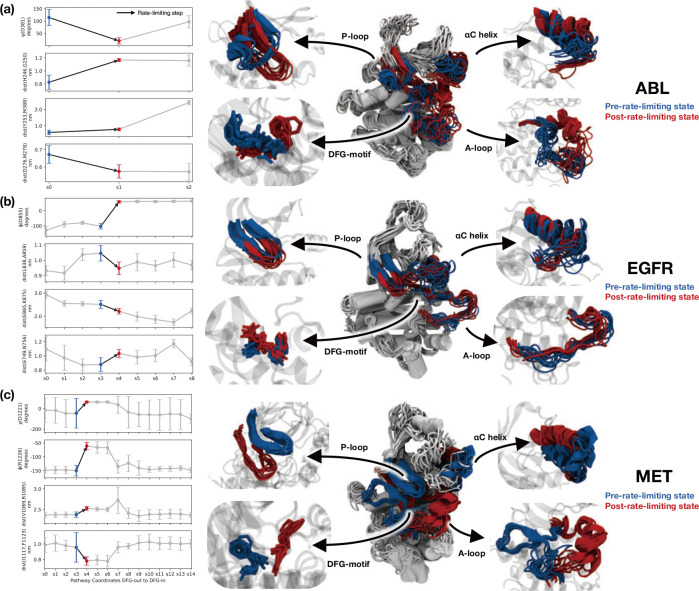
Rate-limiting steps in the DFG-out to DFG-in transition. Analysis for ABL **(a)**, EGFR **(b)**, and MET **(c)**. Left: mean and standard deviation of top-ranked features from the random forest classifier along the transition pathway. The rate-limiting step is indicated by black arrows; pre- and post-rate-limiting states are shown as blue and red markers respectively. Right: superposition of pre-rate-limiting (blue) and post-rate-limiting (red) conformations, overlaid on the full ensemble (grey). Insets highlight key regulatory elements.

While we are simulating the apo form, previous simulation efforts highlight that the MD is capable of capturing key conformational intermediates in activation pathways present across thermal equilibrium (35; 36; 43; 60). We extended these approaches to compute and compare activation pathways and intermediates for ABL, EGFR, and MET kinase domains. To capture activation pathways for ABL, EGFR, and MET, we use using transition path theory (61) by decomposing reactive trajectories from DFG-out (BBAminus) to DFG-in (BLAminus) into reactive fluxes on MSM microstates (Fig. 4). For each kinase domain, we report the highest-flux pathway and define its rate-limiting step as the transition with the smallest flux. Transition path ensembles are provided in Supplementary Fig. S21. To systematically identify structural changes accompanying the rate-limiting step, we trained Random Forest classifiers to distinguish pre- versus post-rate-limiting conformations and extracted the most discriminating structural features (see Methods).

The ABL highest-flux pathway contains two steps, with the BBAminus-to-BLBplus transition being the flux bottleneck (Fig. 4a). The rearrangement is initiated by *α*C helix swinging out, disrupting the *β*3-Lys–*α*C-Glu salt bridge. This opens the back pocket and creates space for the A-loop to rotate towards a SRC-like orientation, an inactive DFG-in conformation, with the two-turn helix packed against the αC helix. Meanwhile, the G-loop transitions from a short helical structure to a more extended turn towards the ATP binding site. The subsequent step transitions from the SRC-like ensemble to the BLAminus ensemble, where the A-loop rotates further to the open conformation, the αC helix returns inward to re-form the salt bridge, and the G-loop returns to a kinked conformation.

The EGFR highest-flux pathway reveals a distinct multi-step transition (Fig. 4b, Supplementary Fig. S21b). It initiates with rearrangement of the X-DFG residue, converting from the DFG-out BBAminus to the inactive DFG-in intermediate ABAminus state. The subsequent ABAminus-to-BLAminus transition is the rate-limiting step, characterized by XDFG motif backbone dihedral reorganization accompanied by subtle A-loop rearrangement, while the A-loop remains open throughout. Minimal structural changes are observed in the G-loop or *α*C helix in the rate-limiting step. Notably, the SRC-like BLBplus ensemble does not participate in the top net-flux pathways, indicating that it forms an off-pathway basin that mostly interconverts with the active-like BLAminus ensemble.

In MET, the highest-flux pathway proceeds from the BBAminus ensemble (state 7) through two BABtrans intermediates (states 1 and 3) before reaching the BLAminus state 6 (Fig. 4c, Supplementary Fig. S21c). The A-loop RMSD to the active state decreases progressively from 21.9 Å (state 7) to 11.4 Å (state 1) and to 3.6 Å (state 3). The rate-limiting transition from state 7 to state 1 is associated with the initial opening of the A-loop, which forms helical elements interacting with the *α*C helix while the DFG motif adopts BABtrans. This is accompanied by *β*3-Lys–*α*C-Glu salt bridge formation and G-loop extension into the space released by A-loop opening. The subsequent transition from state 1 to state 3 involves further opening of the A-loop and dissociation of the helical elements, after which the final transition from state 3 to state 6 completes the BABtrans-to-BLAminus rearrangement. Notably, the path ensemble also shows lower-flux branches connecting both states 1 and 3 directly to the sink state 6, indicating that although the route through states 7, 1, 3, and 6 is dominant, a more direct transition from state 1 to state 6 is also accessible (Supplementary Fig. S21c).

### 2.4 State-dependent activation loop exposure identifies phosphorylation-competent intermediates

The A-loop is a dynamic regulatory element in kinases. Its flexibility allows it to control substrate access via conformational change, but also causes the A-loop to often remain unresolved or partially resolved in experimental crystal structures (62). A-loop phosphorylation provides a common regulatory switch by stabilizing catalytically competent conformations (63), although the conformational state in which phosphorylation sites, sometimes called phosphosites, become accessible can differ between kinases. To examine coupling between phosphorylation site accessibility and A-loop dynamics in our MSMs, we characterized the A-loop ensemble of each macrostate using per-residue secondary-structure labels assigned by DSSP (see Methods) and relative solvent-accessible surface area (rSASA), normalized to a range of 0 to 1 per residue.

**Figure 5:**
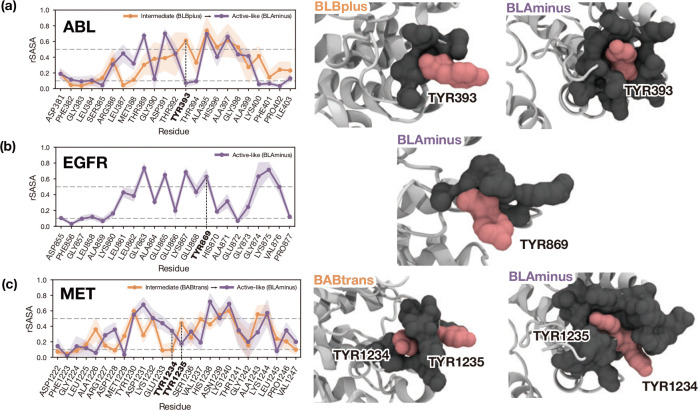
A-loop phosphorylation-site exposure is kinase specific, with Tyr393 (ABL) and Tyr1235 (MET) being more accessible in intermediate ensembles than in the active-like ensemble. Analysis for ABL **(a)**, EGFR **(b)**, and MET **(c)**. rSASA profiles (left column) and representative A-loop conformations (right column) are shown for the active-like ensemble (colored purple, right) and, where active-state (purple) phosphosites are buried, a more exposed intermediate ensemble (colored orange, right). Lines show mean rSASA and shaded regions indicate standard deviation for the sampled conformations; horizontal dashed lines mark rSASA values of 0.1 and 0.5 to indicate complete burial or complete solvent exposure; vertical dashed lines mark phosphosite residues. Phosphosite residues are shown as pink surfaces in the structure. Surrounding residues are shown in grey surfaces.

In the active-like BLAminus ensembles, the phosphosites ABL Tyr393 (63) and MET Tyr1235 (64) show related local packing. DSSP analysis indicates that these sites are embedded in locally structured segments with *β* strands and turns, rather than in fully disordered loops (Supplementary Figs. S18 and S20). In ABL and MET, phosphosite side chains are partially shielded by neighbouring bulky or aliphatic residues, resulting in low solvent exposure in the active-like BLAminus ensemble (Fig. 5a,c).

This state-dependent exposure suggests that the final apo active-like ensemble may not be the main phosphorylation-competent conformation. For MET, this interpretation is consistent with experimental evidence that A-loop phosphorylation is sequential, with Tyr1235 phosphorylated before Tyr1234 (65; 66). In the highest-flux pathway, MET activation proceeds from the BBAminus inactive state through BABtrans state 1 before reaching the active-like BLAminus state 6. In state 1, Tyr1235 is more exposed (rSASA 0.44±0.09), whereas Tyr1234 remains largely buried (rSASA 0.10±0.09). Tyr1234 becomes more exposed only after progression towards the active-like state 6 (rSASA 0.35 ± 0.06). These observations suggest that the BABtrans ensemble may represent a pre-active, phosphorylation-competent intermediate for the first MET A-loop phosphorylation event at Tyr1235, which promotes subsequent phosphorylation at Tyr1234 and A-loop rearrangement towards the active-like ensemble.

The same principle of state-dependent phosphosite accessibility might apply to ABL. Tyr393 is less solvent-exposed in the active-like BLAminus state 5 (rSASA 0.07±0.04) than in the BLBplus ensemble in state 6 (rSASA 0.61±0.09), where Tyr393 points towards solvent (Fig. 5a). This ensemble is also involved in the highest-flux pathway as the post-rate-limiting step. We therefore hypothesize that ABL Tyr393 phosphorylation is preferentially enabled by a BLBplus-like pre-active intermediate that presents Tyr393 in a more solvent-accessible geometry, rather than by the final apo active-like BLAminus state.

EGFR presents a contrasting picture. In the active-like BLAminus ensemble, the A-loop remains more open and predominantly coil-like around Tyr869. The Tyr869 side chain points outward towards solvent and is therefore highly exposed (Fig. 5b). This is consistent with EGFR’s non-canonical regulatory mechanism, in which catalytic activation is primarily mediated by asymmetric kinase-domain dimerisation rather than by a required A-loop phosphorylation switch (67; 68). In this context, Tyr869 exposure may reflect regulatory accessibility for cross-talk phosphorylation rather than a prerequisite step for forming the active catalytic state.

Given these observations from pre-phosphorylation apo ensembles, we hypothesize that A-loop phosphorylation sites are exposed in kinase-specific conformational windows along the activation pathway such that phosphorylation may select or stabilize transient pre-active ensembles rather than marking the final active state. This hypothesis may be tested by combining conformation-selective perturbations with HDX-MS (69), NMR (70; 71), or time-resolved phosphosite mass spectrometry (72) to determine whether phosphosite exposure and phosphorylation order track enrichment of the predicted intermediates.

## 3 Conclusions

Our simulations and MSMs show that kinase activation does not proceed through a single conserved mechanism shared across tyrosine kinases, but through kinase-specific transitions embedded within broader regulatory ensembles. Although ABL, EGFR and MET can access DFG-in, DFG-inter and DFG-out geometries, their equilibrium populations, kinetic connectivity and dominant activation routes differ substantially. Common structural labels can mask such distinct dynamical mechanisms. While the Dunbrack nomenclature provides an interpretable framework for comparing kinase conformations, macrostates with similar DFG assignments can differ in kinetic connectivity. The A-loop is also a major determinant of kinase-specific behaviour, shaping both transition pathways and phosphorylation-site exposure. Together, our results support an ensemble view of kinase activation in which functional states are defined not only by conserved motif geometry, but also by how regulatory elements are thermodynamically populated and kinetically connected.

This mechanistic heterogeneity has direct implications for interpreting kinase regulation and for conformation-selective inhibitor design. The accessibility of DFG-out states, the stability of inactive intermediates, and the coupling between the DFG motif, *α*C helix, G-loop, and A-loop differ between kinases. These features suggest that inhibitor selectivity is shaped by the full conformational network through which active and inactive states are populated and exchanged, rather than by the presence of a nominal DFG-in or DFG-out structure alone. For example, the SRC-like ABL intermediate and the semi-open MET activation-loop ensemble illustrate how transient or sparsely populated states may create kinase-specific opportunities for stabilizing inactive conformations or redirecting activation pathways.

Finally, our work demonstrates how transfer seeding offers a practical route to generate kinase ensembles and build MSMs under a consistent and systematic protocol, even when available experimental structures incompletely cover the relevant conformational landscape. For MET, homologue-guided AF2 models expanded the starting ensemble beyond the available experimentally determined structures and enabled sampling of connected DFG-inter and DFG-out regions. This strategy complements adaptive and enhanced-sampling approaches by using conformational information already present in related family members to initialise unbiased simulations. The present models are limited to isolated apo KDs, while ligand binding, phosphorylation, mutations and regulatory domains may reshape both populations and pathways (29; 30; 31; 73; 74). Ultimately, we envision that extending this framework to perturbed ensembles and linking functional insights with ensemble and kinetic descriptions will open new avenues for the design of next-generation kinase inhibitors with improved selectivity and affinity.

## 4 Methods

### 4.1 Structural seed generation

For each target kinase domain (KD) we assembled a pool of starting conformations (“seeds”) intended to span the DFG-in, DFG-inter, and DFG-out regions of the Modi–Dunbrack *D*_1_ vs. *D*_2_ landscape (14). Seeds were drawn from two sources: (i) experimentally determined structures available in the Protein Data Bank (PDB-based seeding), and (ii) AlphaFold2 (AF2) models generated with ColabFold using curated template sets biased towards specific Dunbrack substates (AF2-based, or “transfer seeding”). All seeds were processed through an identical structure-preparation and solvation pipeline so that downstream simulations differed only in their starting coordinates. Scripts for seed generation are available at https://github.com/sukritsingh/kinase-seeding.

### 4.2 PDB-based seed generation

Apo KD seeds for ABL1 (KLIFS kinase ID 392) and EGFR (KLIFS kinase ID 406) were generated from every structure available for the corresponding kinase in KLIFS (75). For each kinase, the full set of PDB entries, chains, and alternate models was queried with the opencadd KLIFS remote interface and filtered to retain only entries with reported resolution. Each (PDB ID, chain, alternate location) tuple was then passed as a PDBProtein component to the KinoML OEKLIFSKinaseApoFeaturizer (76), which uses the OpenEye Spruce toolkit with the RCSB loop database (rcsb_spruce.loop_db) to strip ligands, waters, and cofactors, model missing loops, repair missing side chains, assign protonation states at pH 7.4, add caps, and return an apo KD. Because the individual structures cover different residue ranges, all apo models for a given kinase were cropped to the common residue window (maximum of the per-structure minimum residue, minimum of the per-structure maximum residue), and any structure with internal gaps in that window was discarded; the retained structures were then re-capped with ACE/NME and residues were renumbered using assign_caps and update_residue_identifiers so that all seeds for a given kinase shared an identical topology. The resulting per-PDB apo structures constitute the PDB-based seeds: 83 seeds for ABL1 and 400 seeds for EGFR.

### 4.3 Transfer seeding of MET via AF2

For MET (KLIFS kinase ID 446, UniProt P08581), the PDB ensemble is dominated by DFG-in conformations. To augment this ensemble, we used homologue-guided AF2 modelling (“transfer seeding”) (51; 52; 77) with MSA subsampling and stochastic dropout enabled. MET KD sequences were modelled using (i) the default ColabFold MSA server (MMseqs2), (ii) stochastic dropout enabled at inference, (iii) num_recycles = 3, and (iv) two models per prediction, to generate a minimal set of AF2 seeds of MET kinase.

Custom template sets derived from the KinCore database (16), sorted by Dunbrack state labels, were used to bias each prediction towards a specific DFG state. The sorted template library is deposited at https://osf.io/spu2a/, and was created by downloading and re-sorting all structures into a directory tree keyed by those labels (one sub-directory per spatial DFG state, and within each, one sub-directory per Dunbrack substate). To bias AF2 towards a chosen state for MET, the templates directory was pointed at the corresponding labelled sub-directory, so that only structures carrying that Dunbrack label were available as templates. Separate AF2 runs were executed for each Dunbrack state template pool. As a minimal set to supplement existing PDB structures, two models from each Dunbrack state were created, giving a total of 16 AF2-based seeds, and were pooled with the native MET PDB structures. This pooled set of 117 PDB-based structures and 16 AF2-seed structures, a total of 133 structures, was then used as the initial MET kinase simulation seeds. The combined set of 133 seeds was processed through the same KinoML apo-kinase featurizer and common-residue cropping described above to yield a single, topology-consistent set of MET seeds. A sample demonstration of using the KinCore-sorted template library to do transfer seeding is available as a Colab notebook at https://colab.research.google.com/drive/1Dcb5ZouYJQfyq1np-TBmKJE8DOAbA9dU.

### 4.4 Molecular dynamics simulations

All seeds (PDB-based for ABL1/EGFR; PDB- and AF2-based for MET) were parameterized and equilibrated with an identical OpenMM 8.0 protocol (78), using AMBER ff14SB for protein (79) and TIP3P for water. Each apo KD was placed in a cubic periodic box with solvent padding on all sides (minimum 1.2 nm) using Modeller.addSolvent, neutralised, and brought to 0.15 M NaCl. To ensure consistent solvation NPT ensembles across conformations, and that conformational differences did not alter the solvent padding, the number of solvent molecules was held consistent at the maximum number of solvent molecules across all conformations. In other words, the largest possible number of solvent molecules designated by the padding cutoff was applied to all conformations. Bonds to hydrogen were constrained, water was held rigid, and hydrogen-mass repartitioning (HMR, hydrogen mass = 4.0 amu) was applied so that a 4 fs Langevin timestep could be used. Nonbonded interactions were treated with PME at a 1.0 nm real-space cutoff. Systems were energy-minimized, then assigned initial velocities from a Maxwell–Boltzmann distribution at 310 K (setVelocitiesToTemperature(310)) and propagated for 5 ns in the NPT ensemble (310 K, 1 atm) with a Monte Carlo Barostat and the Langevin Integrator with a BAOAB-like splitting (collision rate 1.0 ps^−1^, constraint tolerance 10^−5^). These equilibrated structures were launched in full on the Folding@home platform as production runs (37). Production runs were launched with 1000 NPT trajectories per starting structure (“per seed”) using OpenMM 8.0.0 with CUDA mixed precision. Integrator settings, thermostat, barostat, force field, and timestep were identical to equilibration. Example simulation scripts are provided at https://github.com/sukritsingh/distributed-sampling.

### 4.5 DFG conformational state assignment

Simulation snapshots of ABL, EGFR, and MET were assigned to DFG-in, DFG-inter, or DFG-out spatial classes by clustering the *D*_1_ and *D*_2_ distance space using HDBSCAN (80). Each snapshot was then further annotated using the Modi–Dunbrack DFG conformational nomenclature (14). In this scheme, states are labelled as BLAminus, BLAplus, ABAminus, BLBminus, BLBplus, BLBtrans, BABtrans, or BBAminus. The first three letters denote the Ramachandran basins occupied by the backbone dihedrals of the X-DFG, DFG-Asp, and DFG-Phe residues, where X-DFG is the residue immediately preceding the DFG motif. The suffix minus, plus, or trans denotes the $\chi_{1}$ rotamer of DFG-Phe. For each snapshot, a seven-dimensional torsion vector,

$$\theta =\left\{\phi_{\text{X-DFG}},\psi_{\text{X-DFG}},\phi_{\text{DFG-D}},\psi_{\text{DFG-D}},\phi_{\text{DFG-F}},\psi_{\text{DFG-F}},\chi_{1,\;\text{DFG-F}}\right\},$$

was compared with the reference centroid $\theta^{d}$ of each Dunbrack state using the periodic cosine distance

(1) $$D\left(\theta ,\theta^{d}\right)=\frac{2}{7}\sum_{i=1}^{7}\left[1-cos\left(\theta_{i}-\theta_{i}^{d}\right)\right].$$

Snapshots were assigned to the Dunbrack state with the smallest distance. Snapshots for which all state-centroid distances exceeded 1 were classified as noise.

### 4.6 Markov state model construction

Trajectories shorter than 100 ns were discarded. Structural features (see Supplementary Table S2) were extracted, subjected to tICA (lag 100 ns, top 20 components), and discretized into 1000 microstates by *k*-means clustering. Transition counts were computed using effective counts with pseudo-count regularization. Reversible maximum-likelihood MSMs and Bayesian MSMs were estimated using deeptime (81). The choice of MSM parameters was validated by the implied timescale convergence (Supplementary Fig. S7) and kept consistent for ABL, EGFR, and MET. Macrostates were coarse-grained by PCCA+. Reactive pathways were computed using transition path theory (82). Analysis and plotting scripts are available at https://github.com/meyresearch/kinase_analysis.

### 4.7 Enhanced sampling simulations

Accelerated MD (83), Gaussian accelerated MD (84), umbrella sampling (20×20 grid on *D*_1_/*D*_2_), adaptive sampling (85), and metadynamics (86) were deployed on Folding@home and HPC resources targeting MET KD. Reweighting was performed by Miao cumulant expansion (aMD, GaMD) (87), MBAR (umbrella sampling) (88), or summation of Gaussians (in metadynamics) (89). Full details are provided in the Supplementary Methods.

## Supplementary Material

Supplement 1

## Data Availability

All analysis scripts and plotting scripts for MSM construction and analysis are available online at https://github.com/meyresearch/kinase_analysis. MSMs and related models are deposited on Zenodo at https://doi.org/10.5281/zenodo.20544389. The KinCore-sorted template library is hosted at https://osf.io/spu2a/. All scripts for setup and equilibration of seed structures, as well as the trimmed seed structures, are available at https://github.com/sukritsingh/kinase-seeding. Simulation setup and distributed-sampling scripts are available online at https://github.com/sukritsingh/distributed-sampling, including scripts describing simulation parameters for unbiased simulations. Scripts for the framework of adaptive sampling on Folding@home are available at https://github.com/sukritsingh/fahdaptive. The raw MD simulation trajectories for ABL, EGFR and MET comprise approximately 0.5, 1.5 and 2.3 TB of data, respectively. Owing to the size of these datasets and the lack of online storage capacity, the raw trajectories are not deposited in a public repository but are freely available upon any request.
