# Supplementary material for: Tyrosine kinases sample unique activation ensembles
Source: bioRxiv. 2026 Jul 1:2026.06.26.734786. Preprint. [Version 1] doi: 10.64898/2026.06.26.734786 (PMC13345012; doi:10.64898/2026.06.26.734786)
Supplement: Supplement 1 [file media-1.pdf]

Supporting Information: Tyrosine kinases sample unique activation ensembles

Yanchen Zhu, Matteo Degiacomi, Antonia S J S Mey and Sukrit Singh

1 Supplementary methods for enhanced sampling simulations

1.1 Accelerated MD (aMD) on Folding@home

We used Hamelberg-style accelerated MD (1), in which a boost is applied to the total potential energy $V(\mathbf{x})$ whenever $V < E$:

$$V^*(\mathbf{x}) \;=\; V(\mathbf{x}) + \Delta V(\mathbf{x}), \qquad \Delta V(\mathbf{x}) \;=\; \begin{cases} \dfrac{(E - V(\mathbf{x}))^2}{\alpha + E - V(\mathbf{x})}, & V(\mathbf{x}) < E, \\ 0, & V(\mathbf{x}) \geq E, \end{cases} \tag{1}$$

so that forces are scaled by $\partial V^*/\partial V = [\alpha/(\alpha + E - V)]^2$ in the boosted region. The boost parameters (E, α) were chosen from a short (unbiased) calibration run: we recorded $\langle V \rangle$ over the equilibrated trajectory and set $E = \langle V \rangle + \alpha$ with $\alpha = N_{\text{atom}}/5$ for total-potential boosting. An analogous torsion-only variant was also tested (energy and α derived from $\langle V_{\text{tor}} \rangle$ and $3.5\, N_{\text{res}}/5$). The force modification was implemented as a `CustomIntegrator` in OpenMM that performs a standard VRORV Langevin step while replacing $\mathbf{f}$ with $\mathbf{f} \cdot [\alpha/(\alpha + E - V)]^2$ inside the boost region; the ΔV value was stored as an integrator global and dumped at every step for later reweighting (2). One thousand independent aMD replicas were run on the Folding@home from a single apo MET snapshot. Reweighting to the unbiased potential of mean force on (D_1, D_2) was performed with the Miao cumulant expansion up to second order (2).

1.2 Umbrella sampling on Folding@home

The (D_1, D_2) plane was tiled with a 20×20 grid of independent umbrella windows, with centers $(r^0_{1,i}, r^0_{2,j})$ taken from `np.linspace(0.3, 2.0, 20, endpoint=False)` nm on each axis. In each window the system was augmented with two `CustomBondForce` harmonic biases

$$U_{\text{bias}}(\mathrm{D}_1, \mathrm{D}_2) = \tfrac{1}{2}k\,(\mathrm{D}_1 - r^0_{1,i})^2 + \tfrac{1}{2}k\,(\mathrm{D}_2 - r^0_{2,j})^2, \tag{2}$$

with force constant $k = 100$ kJ mol^{-1}nm^{-2} applied between the three atom indices that define D1 and D2. Each window was equilibrated for a short NPT run and then launched as an independent Folding@home work unit using a Langevin integrator (the same VRORV Langevin kernel as above, with ΔV logging disabled). The 2D potential of mean force was obtained by MBAR reweighting over all windows (3).

1.3 Metadynamics

Well-tempered metadynamics (4) was run with PLUMED 2 (5) coupled to OpenMM via the `openmmplumed` plug-in. The bias was deposited along three collective variables: D_1, D_2, and the DFG-Phe $\chi 1$ torsion, with Gaussian height 0.3 kJ mol^{-1} and widths $\sigma = (0.1, 0.1, 0.1)$ in CV units, deposited every 10 integration steps. The simulation was run for 5×10^5 outer iterations of 10 steps each ($\sim$ 10 ns per replica) on a single HPC node. The reweighted free-energy surface was obtained by the Tiwary–Parrinello time-independent estimator (6).

1.4 Gaussian accelerated MD (GaMD)

Gaussian-accelerated MD (7) uses a harmonic boost on the potential energy,

$$\Delta V(\mathbf{x}) \;=\; \begin{cases} \frac{1}{2}\,k\,(E - V(\mathbf{x}))^2, & V(\mathbf{x}) < E, \\ 0, & V(\mathbf{x}) \geq E, \end{cases} \qquad k \;=\; k_0\,\frac{1}{V_{\max} - V_{\min}}, \tag{3}$$

where E is set to the running maximum of V and $k_0 \in (0, 1]$ is tuned so that the standard deviation of ΔV remains below a user-specified bound (typically $\sigma_0 \leq 10\,k_{\mathrm{B}}T$). Under this boost the effective force on coordinate q is $\mathbf{f}^* = \mathbf{f} \cdot [1 - k(E - V)]$, which we again implemented as a `CustomIntegrator` performing VRORV Langevin dynamics with a multiplicative force modifier and per-step logging of ΔV. Boost parameters (E, k) were calibrated following Miao *et al.* (7) from a short preparatory trajectory (initial collection of $V_{\min}/V_{\max}/\langle V\rangle/\sigma_V$, then adaptive update over an "equilibration" phase, then frozen for production). Independent GaMD replicas were launched on Folding@home analogously to aMD. Reweighting on (D_1, D_2) used a cumulant expansion to second order (2).

1.5 Distributed adaptive sampling on Folding@home

For adaptive sampling we used an adaptive seed-controller, building on previous adaptive sampling methods (8): short trajectories are launched in rounds, an initial MSM is estimated after each round, and the next round of seeds is drawn preferentially from microstates that score high on a goal function while retaining an exploration term that up-weights undersampled microstates. Specifically, after each round all currently available MET trajectories were featurized with the DFG-Phe ζ-carbon distance set, projected onto a one-dimensional kinetic-map TICA space (9) (lag 10, dim 1, kinetic_map=`True`), and discretized into $k = 100$ microstates using `pyemma.coordinates.cluster_kmeans`. For each microstate we computed a goal score g_i (here, −RMSD of the microstate centroid to a DFG-out reference) and a count score $c_i = 1/N_i$, with N_i the number of frames currently assigned to microstate i. Both scores were min–max rescaled, and the rank was

$$s_i \;=\; (1 - w)\,\tilde{g}_i + w\,\tilde{c}_i, \tag{4}$$

with explore weight $w = 0.5$ (a companion run used $w = 1.0$, i.e. pure counts-based exploration). Microstates were sorted by s_i and a cumulative-rank cutoff was used to retain the top fraction of ranks from which $n_{\mathrm{seeds}} = 300$ structures were drawn. For each selected frame, a fresh seed was built by deserializing the FAH `system.xml.bz2/state.xml.bz2` reference files, setting the positions and box vectors to those of the chosen frame, drawing new velocities at 310 K, and writing a new `checkpointState.xml.bz2` for the next Folding@home generation. The `next_state.py` worker-side script randomly samples one of the prepared `SEEDS/*.xml.bz2` files per clone.

1.6 Analysis of enhanced sampling simulations

Trajectories from all enhanced-sampling methods were imaged and aligned with `mdtraj` (10). For each frame we computed D_1 and D_2 using the same atom-index triple used by the biasing potentials and, where applicable, the DFG-Phe χ1 torsion. Two-dimensional free-energy surfaces on D_1 vs. D_2 were obtained as follows: for equilibrium seeded MD and adaptive sampling, by direct histogramming with MSM-derived stationary weights; for umbrella sampling, by MBAR reweighting across all 20×20 windows; for aMD and GaMD, by reweighting $\Delta V(t)$ with the Miao second-order cumulant expansion (2; 7); and for metadynamics, by the Tiwary–Parrinello time-independent estimator applied to the deposited Gaussians and the $(\mathrm{D}_1, \mathrm{D}_2)$ colvar trace (6). In all cases the resulting PMF $F(\mathrm{D}_1, \mathrm{D}_2)$ was expressed in $k_{\mathrm{B}}T$ units at 310 K, clipped to a common dynamic range across methods for visualization, and overlaid with the

three Modi–Dunbrack reference centroids (DFG-in, DFG-inter, DFG-out). Effective sample size for the reweighted ensembles was estimated from the normalized reweighting weights w_i as $N_{\text{eff}} = (\sum_i w_i)^2 / \sum_i w_i^2$.

2 Enhanced sampling results for MET kinase

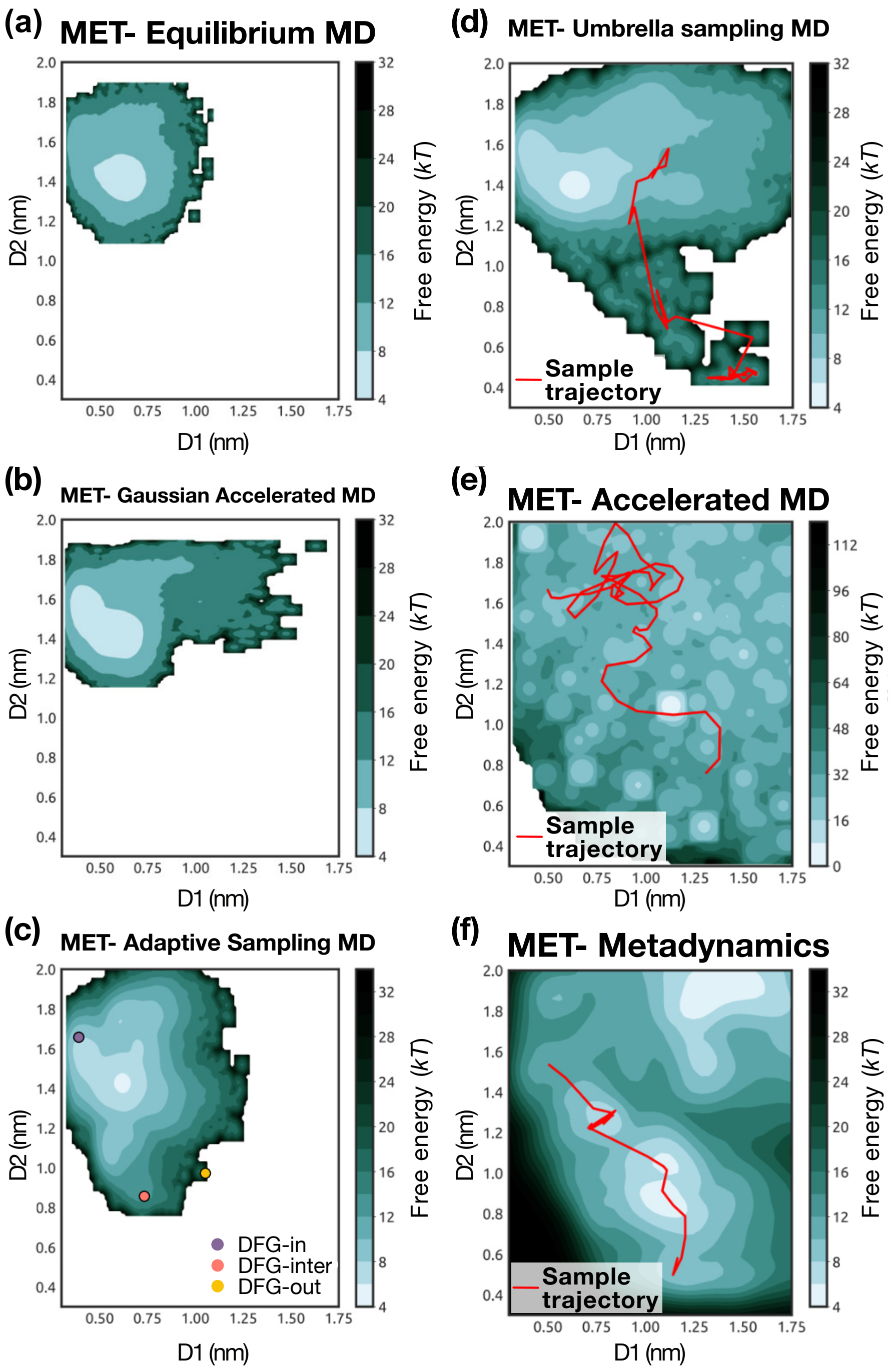

Figure S1: **Comparison of enhanced sampling methods to explore the MET DFG-transition.** Free energy surfaces projected onto D_1 and D_2 from a variety of enhanced sampling method alongside seeded equilibrium MD **(a)**. Enhanced sampling methods used and projected are distributed gaussian Accelerated MD **(b)**, distributed adaptive sampling **(c)**, distributed umbrella sampling **(d)**, distributed accelerated MD **(e)**, and metadynamics **(f)**. For adaptive sampling, representative states that discover the DFG-in (purple), DFG-inter (salmon), and DFG-out (yellow) states are marked (points). For simulations where a transition was observed in a single trajectory, a representative path through D_1 and D_2 space is drawn (red line).

For systems in which the relevant metastable states are not yet known, enhanced-sampling approaches can accelerate barrier crossing and promote exploration of rarely visited conformations. These methods alter the dynamics, energy function, or starting conditions of simulations in controlled ways to explore conformational landscapes more rapidly. Examples include metadynamics, umbrella sampling, accelerated MD (aMD), and Gaussian accelerated MD (GaMD). To avoid biasing the underlying physics that push the system away from equilibrium, adaptive sampling methods instead rapidly prioritize exploration towards an established goal by iterating between simulations from a variety of starting structures, evaluation of the exploration so far, and prioritizing new initial "promising" structures that are more likely to move towards the states of interest. Each of these methods must balance exploration with exploitation tradeoffs to rapidly explore the conformational landscape in trackable ways to recover free energy surfaces.

To quickly and efficiently identify new MET states that bridge the transition between DFG-in and DFG-out states, we deployed four enhanced sampling simulation methods in a distributed manner on the Folding@home platform: aMD, GaMD, umbrella sampling, and adaptive sampling. These approaches are history-independent, modifying only the integrator or the initial structure used for a simulation, and are thus very amenable to massively parallelized dataset generation. In conjunction, we also deployed history-dependent metadynamics simulations that, given a set of collective variables (CVs), explore conformational landscapes using history-dependent biases. This enables us to rapidly generate trajectories and assess how well these methods explore the MET kinase domain structural transition (Supplementary Fig. S1).

Of the methods tested, aMD, umbrella sampling, and metadynamics each achieved complete coverage of the DFG-in, DFG-inter, and DFG-out basins (Supplementary Fig. S1). Replicas of aMD launched from a single DFG-in structure spontaneously discovered DFG-inter and DFG-out conformations without requiring predefined collective variables. Umbrella sampling, tiling the D_1, D_2 plane with a 20×20 grid, provided dense coverage. Well-tempered metadynamics biased along D_1, D_2, and DFG-Phe $\chi 1$ likewise explored all three DFG basins within modest simulation time on a single HPC node. Adaptive sampling discovered the DFG-out basin through iterative rounds of short unbiased trajectories. GaMD did not achieve full coverage, with replicas remaining largely confined to the DFG-in vicinity. Although multiple methods achieved state-space coverage, the resulting free energy surfaces differ substantially across methods (Supplementary Fig. S1). Basin depths, relative free energies, and transition-region topography vary considerably, reflecting differences in bias magnitude, barrier-crossing completeness, and reweighting fidelity. A particularly illustrative case is aMD, which achieved broad state discovery but yielded a free energy surface with pronounced deep minima attributable to low effective sample size (ESS) in the reweighted ensemble. These results highlight that sampling relevant conformational states is necessary but insufficient for quantitative free energy recovery. Taken together, our results above emphasize the challenges associated with enhanced sampling simulations.

3 Additional supporting information

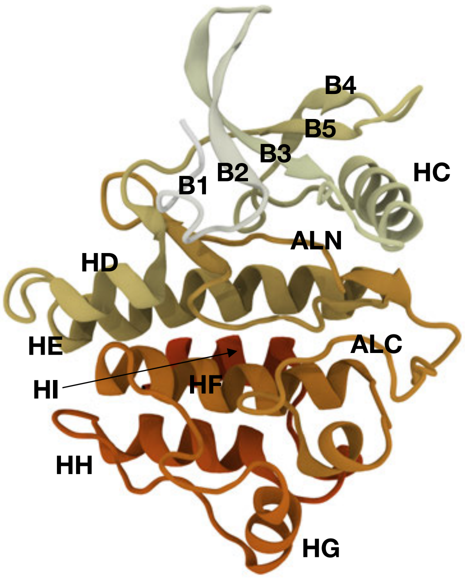

G-loop

	B1	B2	B3	HC	B4
ABL	246-252	253-261	264-274	280-293	294-309
EGFR	712-722	723-731	738-748	756-769	770-784
MET	1076-1088	1089-1097	1103-1113	1121-1134	1135-1150

DFG

	B5	HD	HE	ALN	ALC
ABL	310-320	321-329	334-357	374-394	397-413
EGFR	785-795	796-804	808-831	848-868	872-888
MET	1152-1162	1163-1171	1175-1198	1215-1235	1241-1257

	HF	HG	HH	HI
ABL	414-435	443-455	466-485	486-495
EGFR	889-910	918-930	941-960	961-970
MET	1258-1279	1287-1299	1310-1329	1330-1339

Figure S2: Left, representative ABL kinase domain structure annotated with segment names. Right, residue ranges corresponding to each segment in ABL, EGFR, and MET sequence alignments. Segment names follow the KinCore nomenclature, where **B** denotes β strand, **H** denotes α helix, **ALN** and **ALC** denote the N- and C-terminal portions of the activation loop.

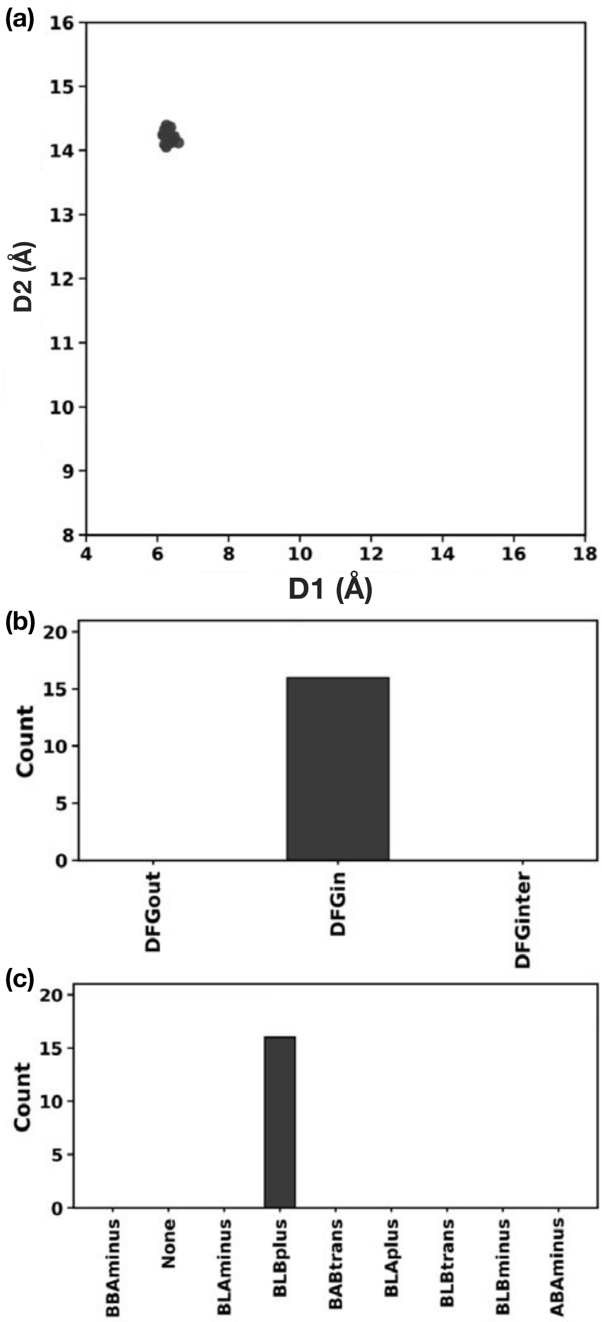

Figure S3: Template-free AlphaFold2 predictions for the MET kinase collapse to a single active-like conformation. **(a)** Two-dimensional projection of AF2 structures of MET without a template along D_1 (F^{DFG}–Cζ to αC+4 residue M1131–Cα) and D_2 (F^{DFG}–Cζ to β3-lysine K1110–Cα). **(b)** Spatial DFG classification of AF2 generated MET structures when no template was provided. **(c)** Dihedral-based Dunbrack DFG-motif classification of each of the generated AF2 conformations without a template.

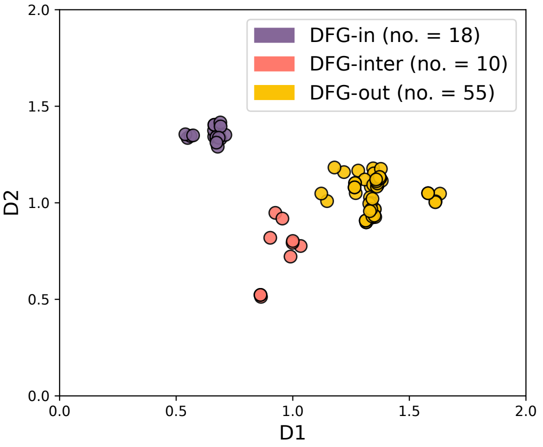

Figure S4: Crystallographic conformational landscape of ABL1 kinase domain (ABL) used as MD seeds. Distribution of all known ABL PDB structures projected onto the DFG spatial coordinates D_1 and D_2 (nm) and classified via KinCore. Each point is a PDB structure coloured by whether it is DFG-in (purple), DFG-out (yellow), or DFG-inter (salmon).

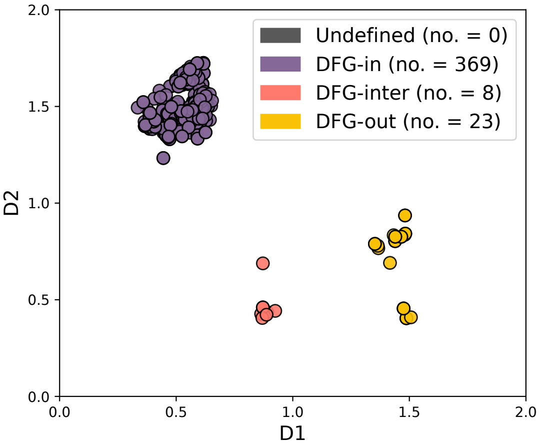

Figure S5: Crystallographic conformational landscape of EGFR kinase used as MD seeds. Distribution of all known EGFR kinase-domain PDB structures projected onto the DFG spatial coordinates D_1 and D_2 (nm) and classified via KinCore. Each point is a PDB coloured by whether the structure is DFG-in (purple), DFG-out (yellow), or DFG-inter (salmon).

Table S1: Kinase PDB IDs used for seeding MD simulations.

Kinase	PDB IDs
ABL	1opl, 2e2b, 2f4j, 2fo0, 2g1t, 2g2f, 2g2h, 2g2i, 2gqg, 2hiw, 2hyy, 2hz0, 2hz4, 2hzi, 2v7a, 3cs9, 3pyy, 3qri, 3qrj, 3qrk, 3ue4, 4twp, 4wa9, 4xey, 4yc8, 4zog, 5hu9, 5mo4, 6bl8, 6npe, 6npu, 6npv
EGFR	1m14, 1m17, 1xkk, 2eb2, 2eb3, 2gs2, 2gs6, 2gs7, 2itn, 2ito, 2itp, 2itq, 2itt, 2itu, 2itv, 2itw, 2itx, 2ity, 2itz, 2j5e, 2j5f, 2j6m, 2jit, 2jiu, 2jiv, 2rf9, 2rfd, 2rfe, 2rgp, 3bel, 3gop, 3gt8, 3ika, 3lzb, 3poz, 3ug1, 3ug2, 3vjn, 3vjo, 3w2o, 3w2p, 3w2q, 3w2r, 3w2s, 3w32, 3w33, 4g5j, 4g5p, 4hjo, 4i1z, 4i20, 4i21, 4i22, 4i23, 4i24, 4jq7, 4jq8, 4jr3, 4jrv, 4li5, 4ll0, 4lqm, 4lrm, 4r3p, 4r3r, 4r5s, 4riw, 4rix, 4riy, 4rj4, 4rj5, 4rj6, 4rj7, 4rj8, 4tks, 4wd5, 4wkq, 4wrg, 4zau, 4zjv, 4zse, 5c8k, 5c8m, 5c8n, 5cal, 5can, 5cao, 5cap, 5caq, 5cas, 5cau, 5cav, 5cnn, 5cno, 5czh, 5czi, 5d41, 5edp, 5edq, 5edr, 5em5, 5em6, 5em7, 5em8, 5fed, 5fee, 5feq, 5gmp, 5gnk, 5gty, 5gtz, 5hcx, 5hcy, 5hcz, 5hg5, 5hg7, 5hg8, 5hg9, 5hib, 5hic, 5j9y, 5j9z, 5jeb, 5u8l, 5uga, 5ugb, 5ugc, 5uwd, 5x2a, 5x2c, 5x2f, 5x2k, 5x26, 5x27, 5x28, 5xdk, 5xdl, 5xgm, 5xgn, 5y9t, 5y25, 5yu9, 5zto, 5zwj, 6d8e, 6duk, 6jrj, 6jrk, 6jrx, 6jwl, 6jx0, 6jx4, 6jxt, 6jz0, 6lub, 6lud, 6p1d, 6p1l, 6p8q, 6s8a, 6s9b, 6s9c, 6s9d, 6s89, 6tfu, 6tfv, 6tfw, 6tfy, 6tfz, 6tg0, 6tg1, 6v5n, 6v5p, 6v6k, 6v6o, 6v66, 6vh4, 6vhn, 6vhp, 6wa2, 6wak, 6z4b, 6z4d, 7a2a, 7aem
MET	1r0p, 1r1w, 2g15, 2rfs, 2wd1, 2wgj, 2wkm, 3a4p, 3ccn, 3cd8, 3ce3, 3cth, 3dkc, 3dkf, 3dkg, 3f66, 3i5n, 3l8v, 3lq8, 3q6w, 3qti, 3r7o, 3rhk, 3vw8, 3zbx, 3zc5, 3zcl, 3zxz, 3zze, 4aoi, 4ap7, 4deg, 4deh, 4dei, 4gg5, 4gg7, 4iwd, 4knb, 4mxc, 4r1v, 4r1y, 4xmo, 4xyf, 5dg5, 5eob, 5eyc, 5eyd, 5hlw, 5hni, 5ho6, 5hor, 5hti, 5uab, 5uad, 5uaf, 5ya5, 6sd9, 6sdc, 6sdd, 6sde, 6ubw, 7b3q, 7b3t, 7b3v, 7b3w, 7b3z, 7b40, 7b41, 7b42, 7b43, 7b44

Table S2: Selected features of ABL, EGFR, and MET for MSM construction.

Feature	Description	Dimensions
DFG distances	The distances of DFG-Phe-Cζ from (i) αC-Met-Cα and (ii) β3-Lys-Cα	2
DFG dihedrals	ϕ and ψ angles of the X-DFG, DFG-Asp, and DFG-Phe, and χ1 angle of DFG-Phe converted to sin and cos values	18
αC helix distances	The distances of αC-Glu-Cδ from (i) β3-Lys-Nζ and (ii) A-loop Arg386 (ABL), Arg1227 (MET), or Lys860 (EGFR)	2
A-loop distances	Pairwise Cα distances separated by at least two residues between Asp381–Ile403 (ABL), Asp855–Asp877 (EGFR), or Asp1222–Val1247 (MET)	210 (ABL)<br>210 (EGFR)<br>276 (MET)

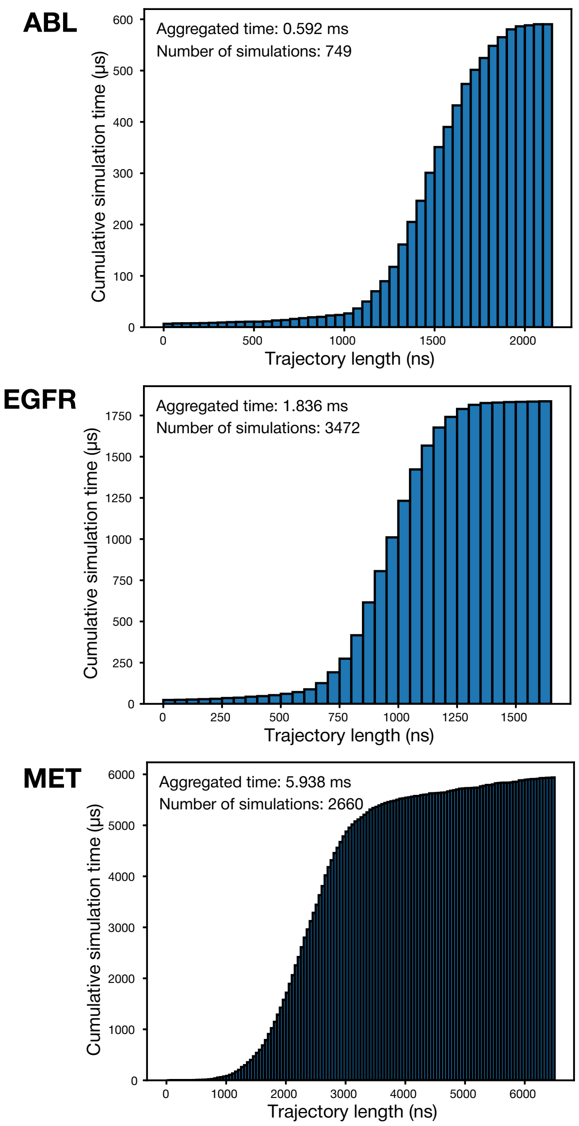

Figure S6: Cumulative simulation time for ABL, EGFR, and MET shown as a function of trajectory length binned in 50 ns.

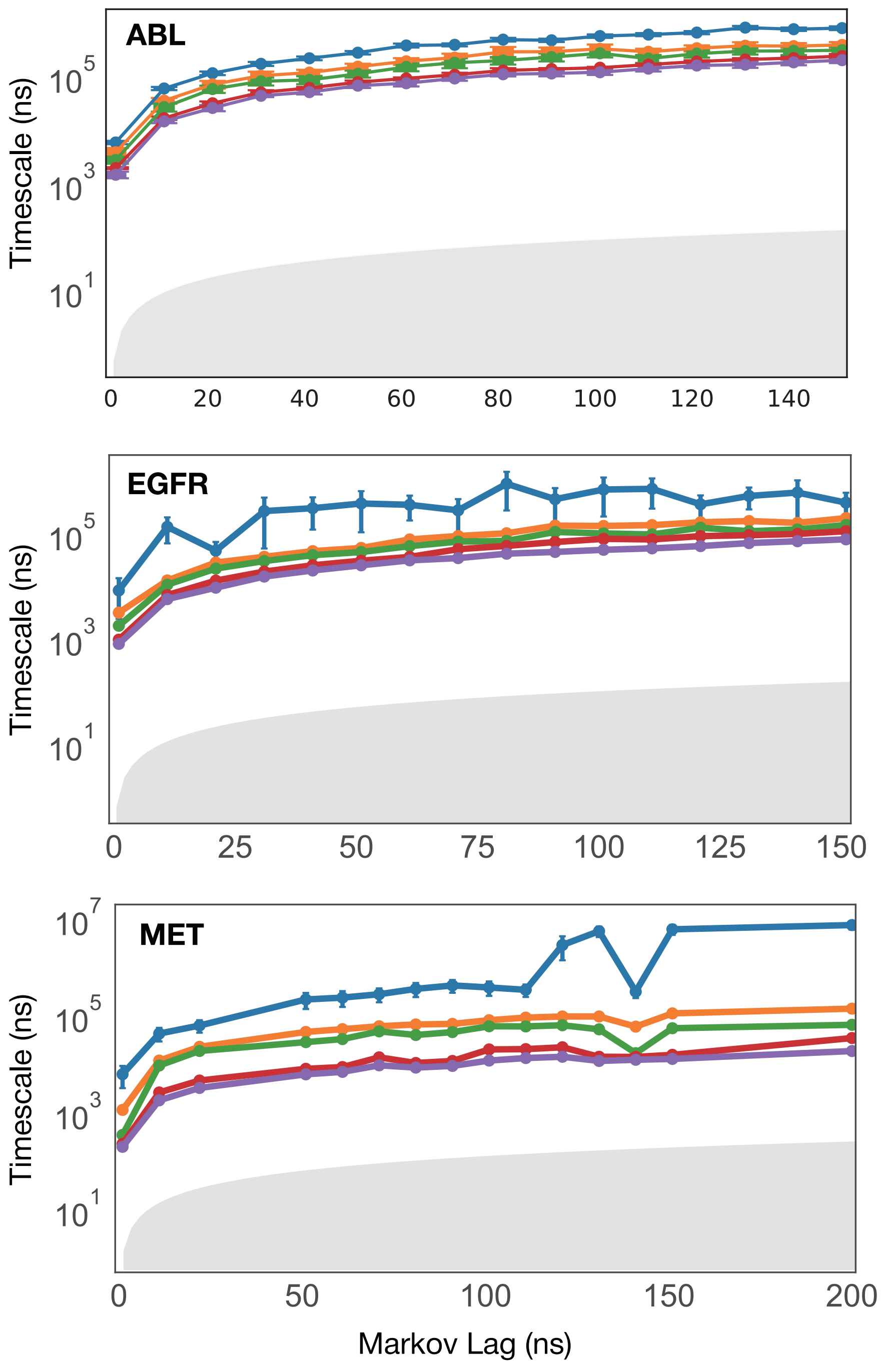

Figure S7: The five slowest timescales for ABL, EGFR, and MET estimated at Markov lag times from 1 ns to 151 ns in increments of 10 ns. The shaded area indicates the identity of timescales. The error bars show the standard deviation derived from Bayesian MSMs.

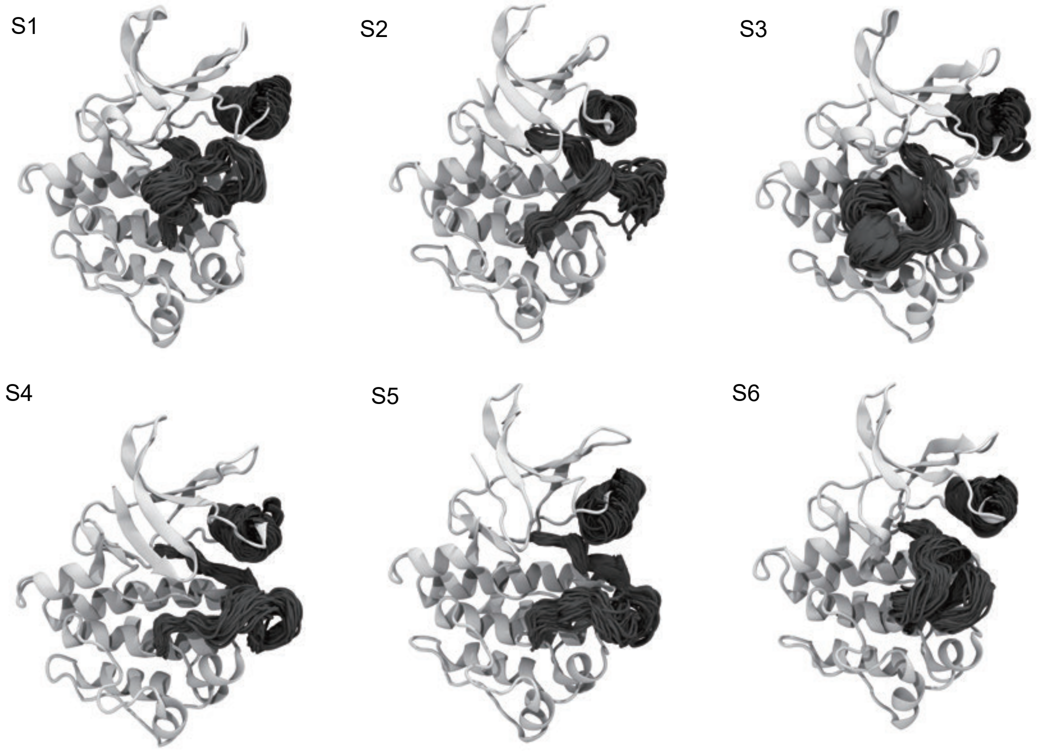

Figure S8: A-loop and αC helix (shaded regions) structural ensembles for ABL macrostates. The corresponding macrostate is indicated (top left above each structure).

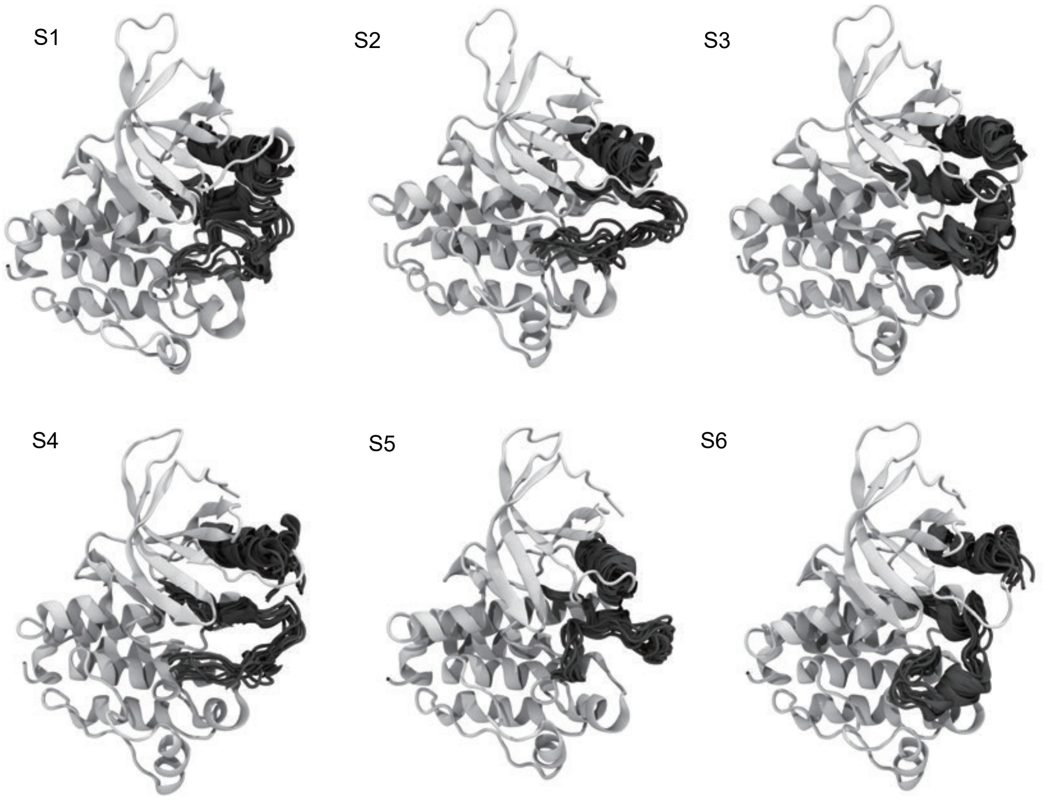

Figure S9: A-loop and αC helix structural ensembles for EGFR macrostates. The corresponding macrostate is indicated (top left above each structure).

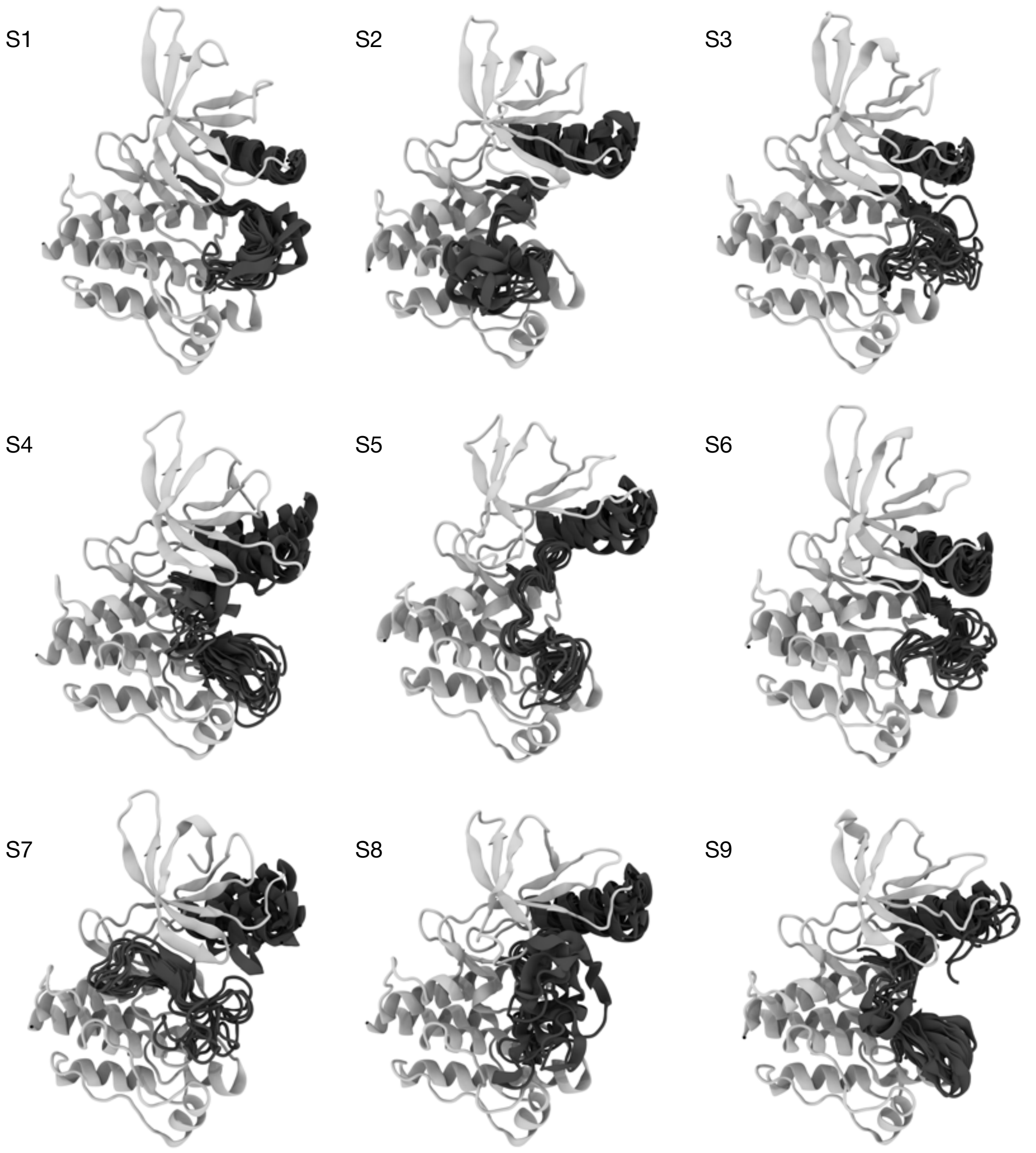

Figure S10: A-loop and αC helix structural ensembles for MET macrostates. The corresponding macrostate is indicated (top left above each structure)

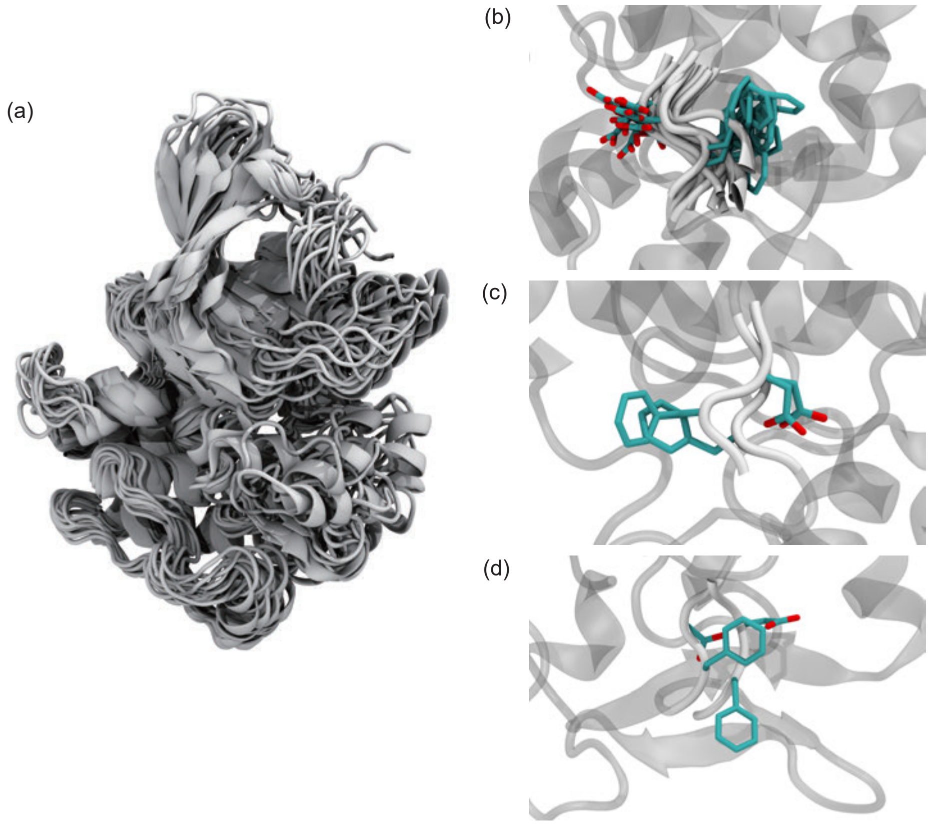

Figure S11: AF2 seeded MET conformations in different Dunbrack states derived using transfer seeding. (a) Structural overlay of all 16 seeded conformations. (b, c, d) The DFG motifs of structures seeded in DFG-in (b), DFG-inter (c), and DFG-out (d).

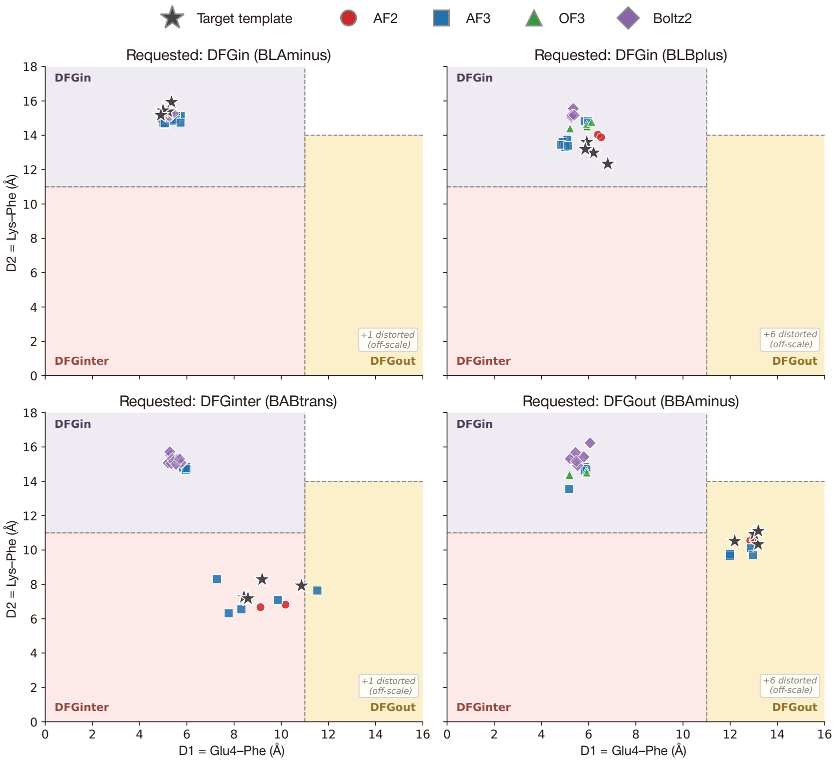

Figure S12: MET structures predicted using transfer-seeding with a variety of co-folding models given a set of templates (gray star). All structures are projected onto the same D_1 vs. D_2 access for comparison for one of four given templates: BLAminus (top left), BLBplus (top right), BABtrans (bottom left), and BBAminus (bottom right). Co-folding models tested were AlphaFold2 (red circle), AlphaFold3 (blue squre), OpenFold3 (green triangle), and Boltz-2 (purple diamond), each of which were provided with the same structural templates (gray star).

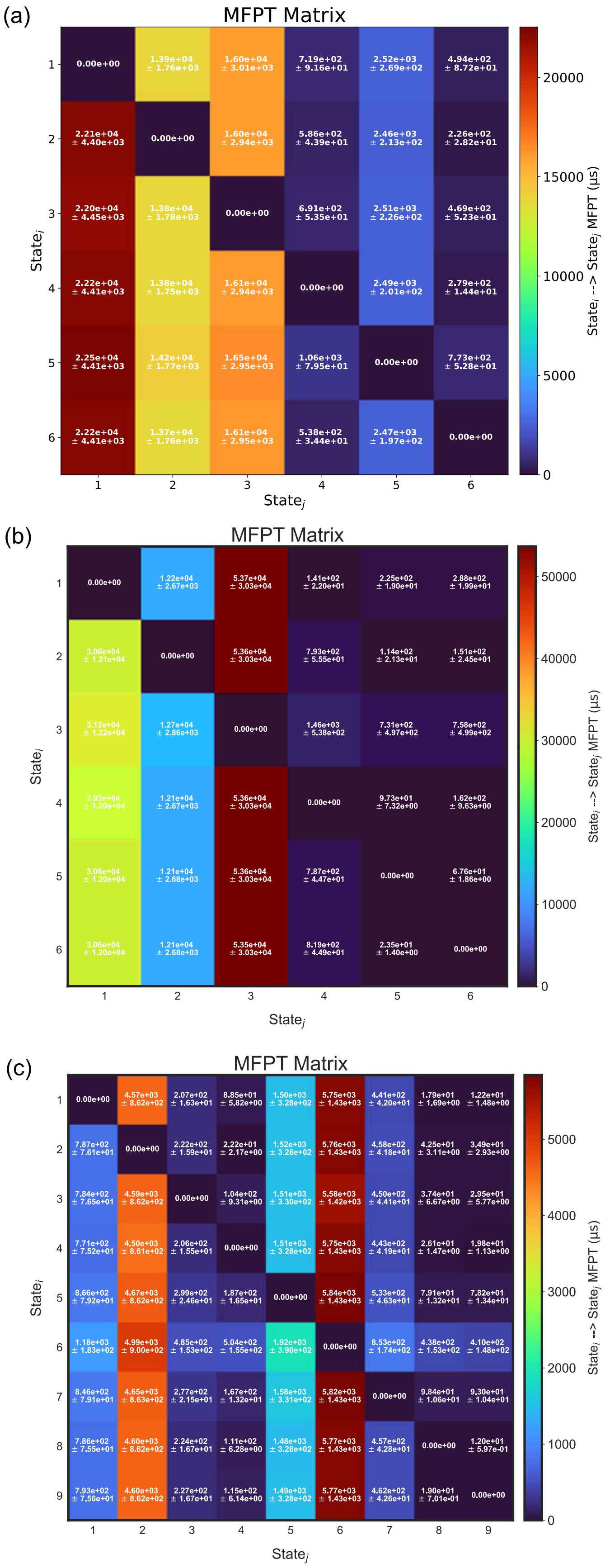

Figure S13: The mean first passage times between metastable macrostates for (a) ABL, (b) EGFR, and (c) MET. The color of the heatmap is indicative of the MFPT (colorbar, right).

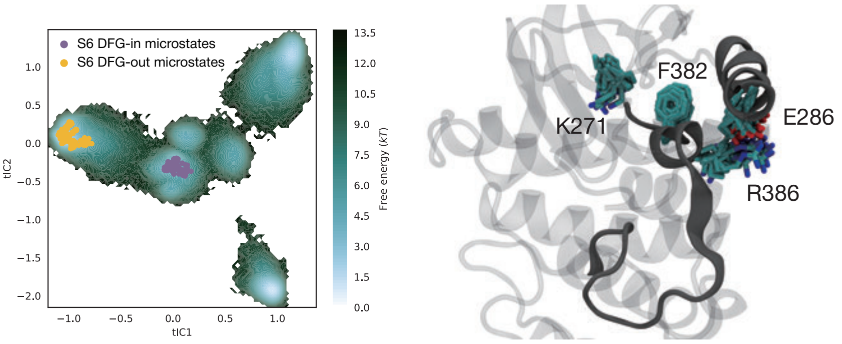

Figure S14: Projecting microstates that represent ABL microstate 6.The DFG-in BLBplus and DFG-out BBAminus microstates of ABL macrostate 6 (left). A zoomed-in view of the A-loop-Arg-αC-Glu salt bridge of the DFG-in BLBplus (SRC-like inactive) substate (right).

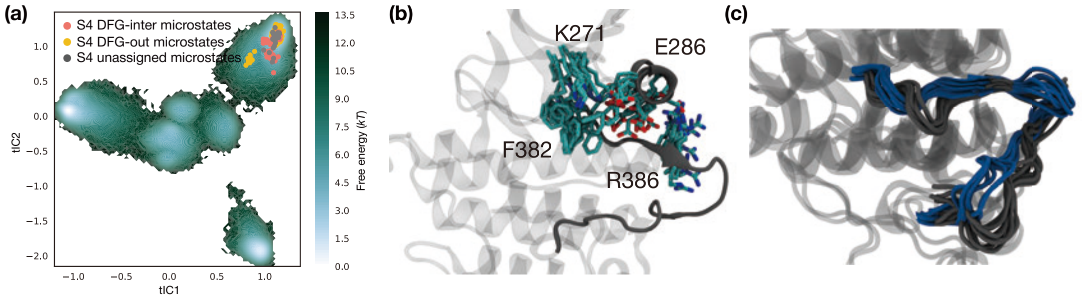

Figure S15: The microstates that make up ABL macrostate 4(a) The DFG-inter BABtrans, DFG-out BBAminus, and unassigned microstates of ABL macrostate 4. (b) A zoomed-in view of the intermittently formed β3-Lys αC-Glu salt bridge of ABL macrostate 4. (c) Comparison of ABL A-loop conformations between macrostate 4 (blue) and macrostate 5 (grey).

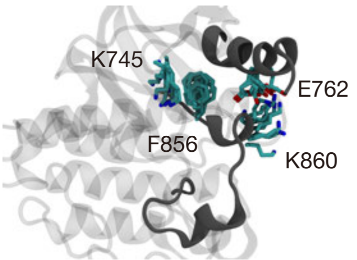

Figure S16: A zoomed-in view of the A-loop-Lys αC-Glu salt bridge of the EGFR SRC-like inactive macrostate 6.

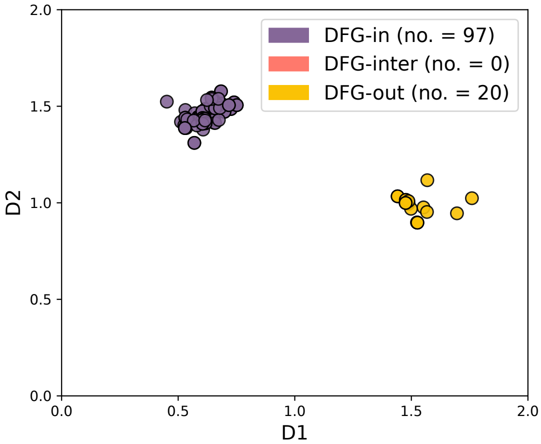

Figure S17: Crystallographic conformational landscape of MET kinase used as MD seeds. Distribution of all known MET kinase-domain PDB structures projected onto the DFG spatial coordinates D_1 and D_2 (nm) as classified via KinCore. Each point is a PDB coloured by whether the structure is DFG-in (purple), DFG-out (yellow), or DFG-inter (salmon).

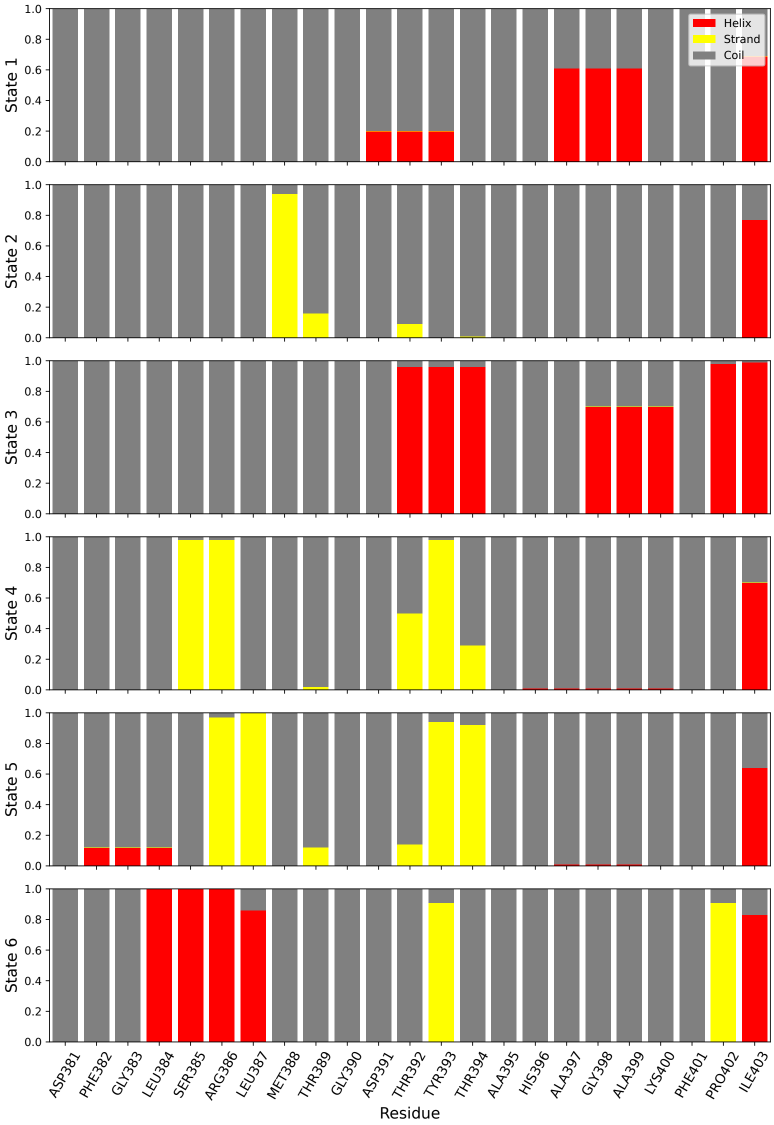

Figure S18: Secondary structure propensities of ABL A-loop subset (Asp381–Ile403) across each of the six macrostates, showing the fractional occupancy of DSSP-assigned elements for each residue. For each state (rows), each residue (column) is classified for the proportion of time spent as a helix (red), a coil (gray) or a β-strand (yellow).

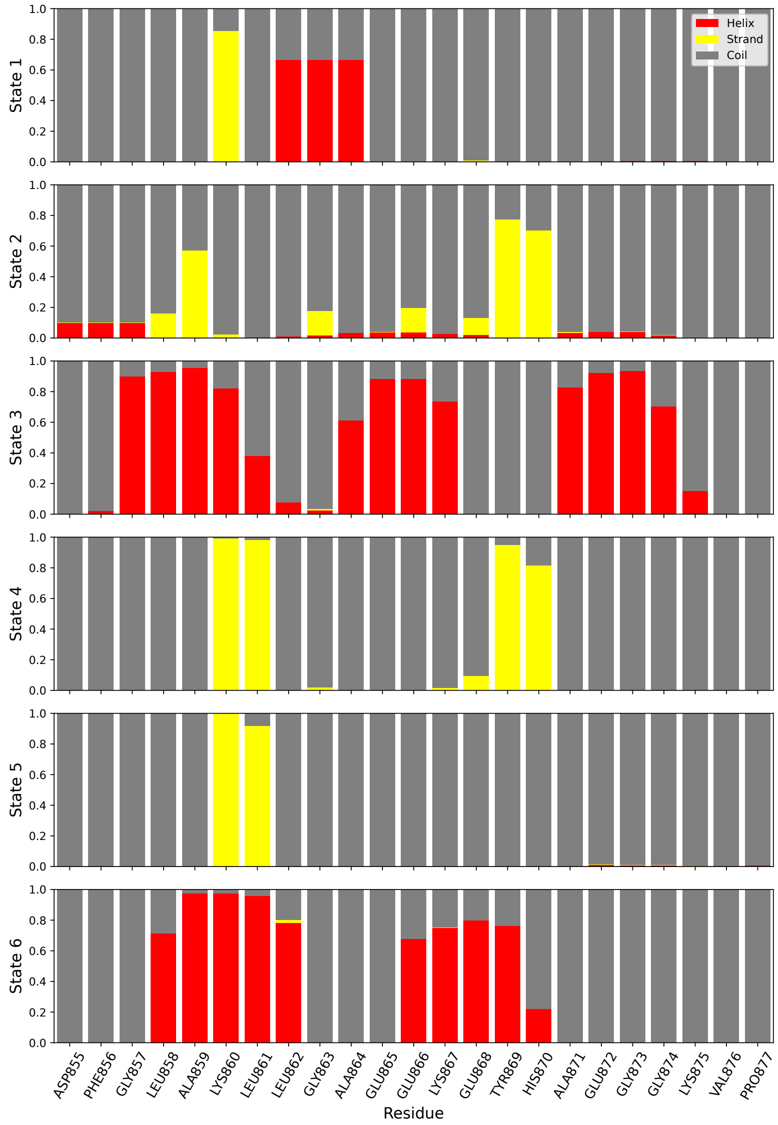

Figure S19: Secondary structure propensities of EGFR A-loop subset (ASP855–PRO877) across each macrostates, showing the fractional occupancy of DSSP-assigned elements for each residue. For each state (rows), each residue (column) is classified for the proportion of time spent as a helix (red), a coil (gray) or a β-strand (yellow).

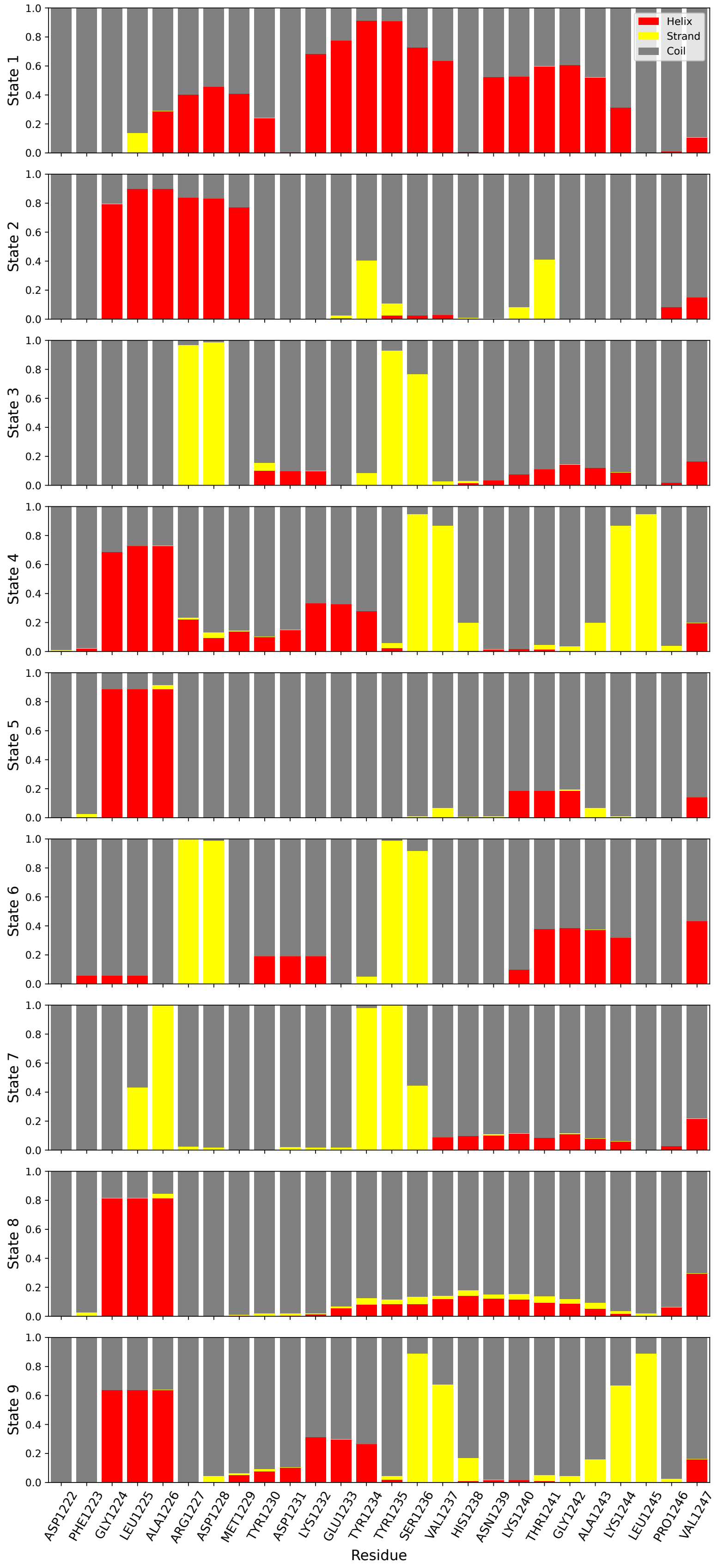

Figure S20: Secondary structure propensities of MET A-loop subset (Asp1222–Val1247) across each macrostate, showing the fractional occupancy of DSSP-assigned elements for each residue. For each state (rows), each residue (column) is classified for the proportion of time spent as a helix (red), a coil (gray) or a β-strand (yellow).

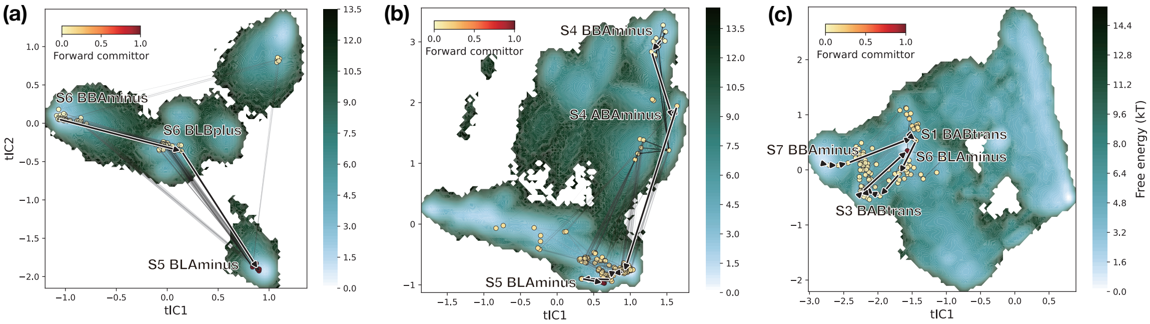

Figure S21: The top 100 highest flux pathways of (a) ABL, (b) EGFR, and (c) MET transitioning from a dominant BBAminus to BLAminus ensemble. Each line's thickness is proportional to its net transition flux. The highest flux pathway is highlighted (outlined arrows). The relevant microstates are shown and coloured by their forward committor probabilities (colorbar, top left).

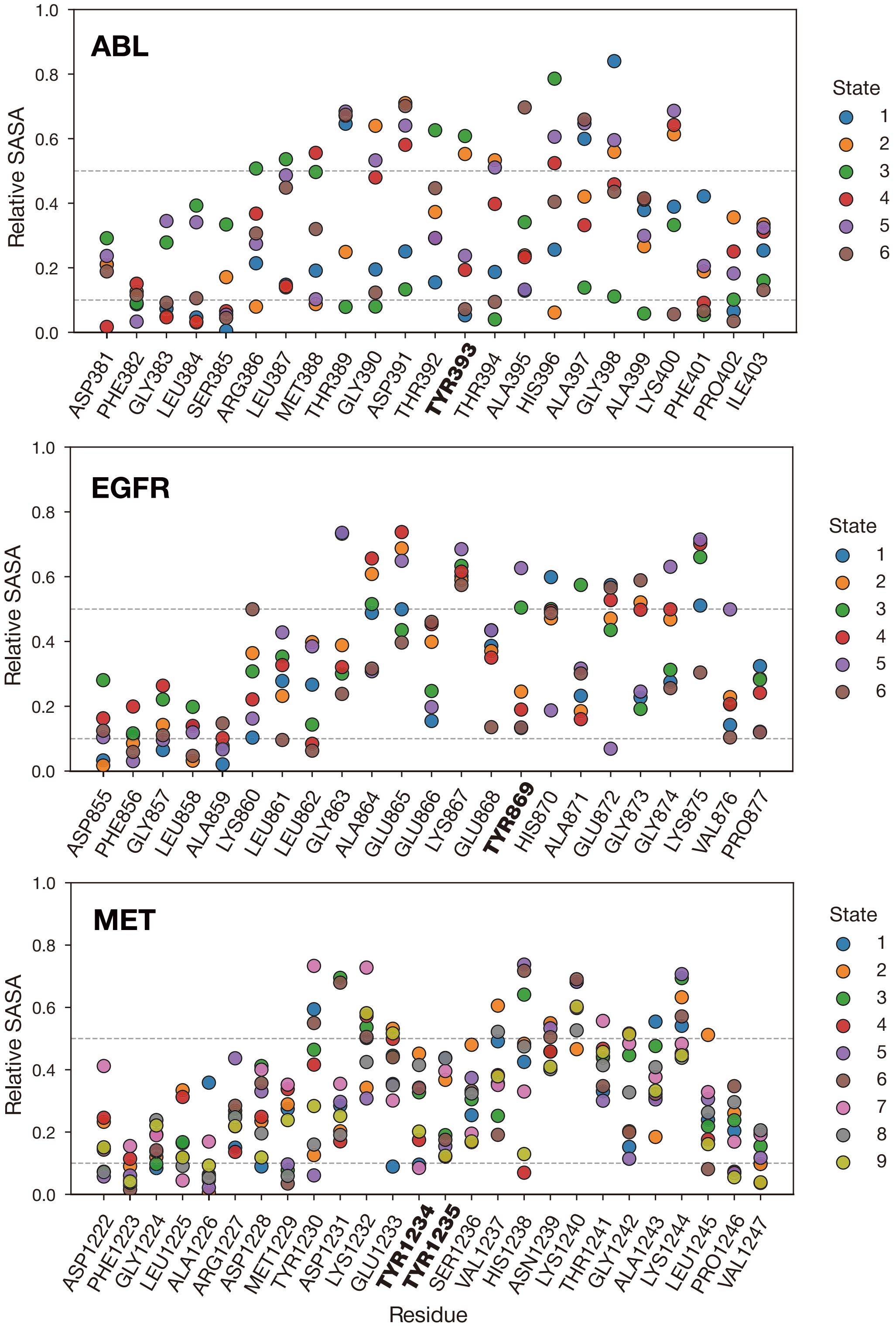

Figure S22: Residue-wise relative solvent-accessible surface area (rSASA) of the activation loops for ABL (top), EGFR (middle), and MET (bottom). Absolute SASA values were normalized by the residue-specific maximum accessible surface areas reported by Tien et al. (11), and per-residue rSASA values are reported per state (colors, right). Grey dashed lines correspond to relative SASA values of 0.1 and 0.5 to indicate complete burial or complete solvent exposure. Tyrosine phosphorylation sites reported over 50 times in PhosphoSitePlus are highlighted in bold (12).

Supplementary References

[1] Hamelberg, D., Mongan, J. & McCammon, J. A. Accelerated molecular dynamics: A promising and efficient simulation method for biomolecules. *The Journal of Chemical Physics* **120**, 11919–11929 (2004). URL https://doi.org/10.1063/1.1755656.

[2] Miao, Y. *et al.* Improved Reweighting of Accelerated Molecular Dynamics Simulations for Free Energy Calculation. *Journal of Chemical Theory and Computation* **10**, 2677–2689 (2014). URL https://doi.org/10.1021/ct500090q.

[3] Shirts, M. R. & Chodera, J. D. Statistically optimal analysis of samples from multiple equilibrium states. *The Journal of Chemical Physics* **129**, 124105 (2008). URL https://www.ncbi.nlm.nih.gov/pmc/articles/PMC2671659/.

[4] Laio, A. & Parrinello, M. Escaping free-energy minima. *Proceedings of the National Academy of Sciences* **99**, 12562–12566 (2002). URL http://www.pnas.org/content/99/20/12562.full.

[5] Tribello, G. A., Bonomi, M., Branduardi, D., Camilloni, C. & Bussi, G. PLUMED 2: New feathers for an old bird. *Computer Physics Communications* **185**, 604–613 (2014). URL http://linkinghub.elsevier.com/retrieve/pii/S0010465513003196.

[6] Tiwary, P. & Parrinello, M. A time-independent free energy estimator for metadynamics. *The Journal of Physical Chemistry B* **119**, 736–742 (2015). URL https://doi.org/10.1021/jp504920s. PMID: 25046020, https://doi.org/10.1021/jp504920s.

[7] Miao, Y., Feher, V. A. & McCammon, J. A. Gaussian Accelerated Molecular Dynamics: Unconstrained Enhanced Sampling and Free Energy Calculation. *Journal of Chemical Theory and Computation* **11**, 3584–3595 (2015). URL https://pubs.acs.org/doi/10.1021/acs.jctc.5b00436.

[8] Zimmerman, M. I. & Bowman, G. R. FAST Conformational Searches by Balancing Exploration/Exploitation Trade-Offs. *J. Chem. Theory Comput.* **11**, 5747–5757 (2015).

[9] Scherer, M. K. *et al.* PyEMMA 2: A Software Package for Estimation, Validation, and Analysis of Markov Models. *Journal of Chemical Theory and Computation* **11**, 5525–5542 (2015). URL https://doi.org/10.1021/acs.jctc.5b00743.

[10] McGibbon, R. T. *et al.* MDTraj: A Modern Open Library for the Analysis of Molecular Dynamics Trajectories. *Biophysical Journal* **109**, 1528–1532 (2015). URL http://linkinghub.elsevier.com/retrieve/pii/S0006349515008267.

[11] Tien, M. Z., Meyer, A. G., Sydykova, D. K., Spielman, S. J. & Wilke, C. O. Maximum Allowed Solvent Accessibilites of Residues in Proteins. *PLOS ONE* **8**, e80635 (2013). URL https://journals.plos.org/plosone/article?id=10.1371/journal.pone.0080635.

[12] Hornbeck, P. V. *et al.* PhosphoSitePlus, 2014: mutations, PTMs and recalibrations. *Nucleic Acids Research* **43**, D512–D520 (2015). URL https://doi.org/10.1093/nar/gku1267.
